# Structural insights into the mechanotransducing mechanism of FtsEX in cell division

**DOI:** 10.1002/mco2.688

**Published:** 2024-10-20

**Authors:** Yuejia Chen, Du Guo, Xin Wang, Changbin Zhang, Yatian Chen, Qinghua Luo, Yujiao Chen, Lili Yang, Zhibo Zhang, Tian Hong, Zhengyu Zhang, Haohao Dong, Shenghai Chang, Jianping Hu, Xiaodi Tang

**Affiliations:** ^1^ Department of Laboratory Medicine, State Key Laboratory of Biotherapy, National Clinical Research Center for Geriatrics, West China Hospital Sichuan University Chengdu China; ^2^ Frontiers Medical Center, Tianfu Jincheng Laboratory, West China Hospital Sichuan University Chengdu China; ^3^ Department of Clinical Laboratory, Zhongnan Hospital of Wuhan University, School of Pharmaceutical Sciences Wuhan University Wuhan China; ^4^ Center of Cryo‐Electron Microscopy Zhejiang University Hangzhou Zhejiang China; ^5^ Key Laboratory of Medicinal and Edible Plants Resources Development of Sichuan Education Department, College of Pharmacy and Biological Engineering, Sichuan Industrial Institute of Antibiotics Chengdu University Chengdu China

**Keywords:** ABC transporter, cell division, EnvC, FtsEX, mechanotransmission

## Abstract

The filamentous temperature‐sensitive (Fts) protein FtsEX plays a pivotal role in *Escherichia coli* (*E. coli*) cell division by facilitating the activation of peptidoglycan hydrolysis through the adaptor EnvC. FtsEX belongs to the type VII ATP‐binding cassette (ABC) transporter superfamily, which harnesses ATP energy to induce mechanical force, triggering a cascade of conformational changes that activate the pathway. However, the precise mechanism by which FtsEX initiates mechanotransmission remains elusive. Due to the inherent instability of this type of ABC transporter protein in vitro, the conformation of FtsEX has solely been determined in the stabilized ATP‐bound state. To elucidate the dynamics of FtsEX, we characterized FtsEX and EnvC of various functional structures through cryo‐electron microscopy (cryo‐EM) and homology modeling. We validated the structures by molecular dynamics simulations. By site‐directed mutagenesis and phenotype screening, we also identified the functional residues involved in allosteric communication between FtsE and FtsX as well as FtsX and EnvC. Additionally, we discovered a potential role of phospholipids in stabilizing the complex conformation during mechanotransmission. This comprehensive exploration significantly enhances our understanding of the intricate mechanisms governing bacterial cell division and unveils potential molecular targets for developing innovative antimicrobial drugs to combat antibiotic resistance.

## Graphical abstract

FtsEX plays a pivotal role in cell division by facilitating the activation of peptidoglycan hydrolysis through the adaptor EnvC. By characterizing the structures of FtsEX and EnvC in different states using cryo‐electron microscopy (cryo‐EM) and homology modeling, we propose a mechanotransducing mechanism for FtsEX: ATP binding brings the FtsEX dimer close together, inducing a rotary shift in the periplasmic loop domains (PLDs), which results in activating conformational changes in EnvC.

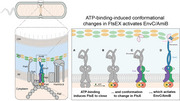

## 1 INTRODUCTION

**FIGURE 1:**
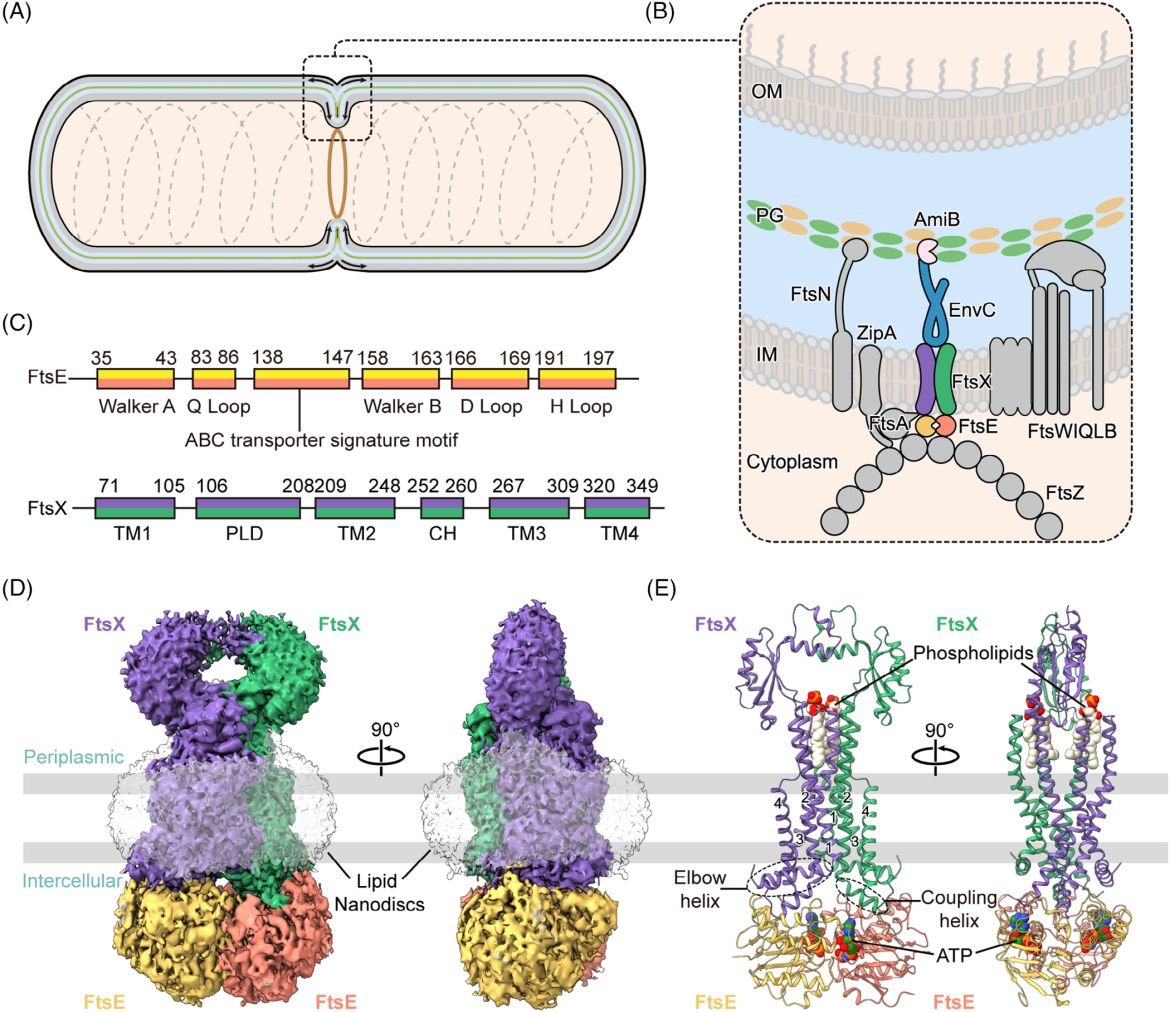
Structural characterization of the FtsE(E163Q)X complex from *Escherichia coli* (*E. coli*). (A, B) Schematic diagram of peptidoglycan hydrolysis at the septum by the divisome. The FtsEX, EnvC, and AmiB are highlighted in bright colors. (C) Topological structures of FtsE and FtsX. (D) cryogenic electron microscopy (cryo‐EM) density map of FtsEX complex. (E) Cartoon representation of the atomic models of FtsEX complex with the binding of ATP and phospholipids. FtsE is in gold and salmon and FtsX is in green and purple.

Antimicrobial resistance poses a threat to global public health.^1^, ^2^ The composition and structure of the bacterial cell wall are crucial factors in determining antibiotic susceptibility.^3^, ^4^, ^5^ Peptidoglycan (PG) is the major structural component of the cell wall. Its synthesis and hydrolysis are tightly controlled during bacterial cell division.^6^ Gram‐negative bacteria undergo a two‐step division process primarily governed by a large protein complex called the divisome^7^, ^8^ (Figure 1A,B). A mid‐cell septum formation involves the initiation of a Z‐ring assembled at the inner membrane, which is mediated by a highly conserved microtubule protein analog FtsZ.^9^ When the Z‐ring is formed, two membrane‐anchoring proteins, ZipA and FtsA, bind to the conserved C‐terminal peptide (CCTP) of FtsZ, mediating their anchoring to the inner membrane.^10^ Subsequently, FtsEX arrives at the septum and interacts with the Z‐ring, coordinating the recruitment of other cell division‐associated proteins, including FtsQLB, FtsWI, and FtsN.^7^, ^11^ After that, the divisome matures and induces cell contraction and septal peptidoglycan (sPG) synthesis^12^, ^13^ (Figure 1A,B). The newly formed sPG layer must be promptly divided to separate the daughter cells.^14^ In *Escherichia coli* (*E. coli*), sPG hydrolysis is mediated by the PG amidase AmiB, and its activity is tightly regulated by FtsEX–EnvC. This highly orchestrated process ensures the successful division of bacterial cells.^7^, ^15^, ^16^ Disrupting this process leads to bacterial proliferation arrest, making it a suitable target for antimicrobial drug development.

FtsEX is a member of the type VII ATP‐binding cassette (ABC) transporter family, which also includes the tripartite efflux pump protein MacB and the lipoprotein transporter LolCDE.^17^, ^18^, ^19^, ^20^ FtsX is an integral membrane protein, constituting the transmembrane domains (TMDs) and periplasmic loop domains (PLDs),^17^, ^21^ while FtsE is a cytoplasmic ATPase, constituting the nucleotide‐binding domains (NBDs) (Figure 1C). Typically in bacterial ABC transporters, ATPase activity of NBDs prompts conformational changes within the TMDs and PLDs, enabling substrate translocation across the membrane.^22^ However, FtsEX does not contain an intrinsic substrate, and it primarily functions as a mechanotransducer, inducing conformational changes in the adaptor protein EnvC to activate the PG amidase AmiB.^6^, ^23^, ^24^, ^25^, ^26^

Recent studies on the structure of FtsEX from *Pseudomonas aeruginosa*, *Vibrio cholerae*, and *Mycobacterium tuberculosis* have revealed the molecular details of FtsEX.^21^, ^27^, ^28^ FtsEX demonstrates high configurational homology to other type VII ABC transporters and a high degree of conservation across different bacterial species^29^ (Figure S1A). However, due to the inherent instability of the apo form of the type VII ABC transporter proteins in vitro, these reported structures are primarily from ATP‐stabilized complex, exhibiting an NBDs‐dimerized conformation. To elucidate the conformational dynamics of FtsEX, we acquired the cryogenic electron microscopy (cryo‐EM) structures of ATP‐bound FtsEX and FtsEX–EnvC from *E. coli* by cryo‐EM. We also adopted the apo state of FtsEX or FtsEX–EnvC through homology modeling, AlphaFold prediction, and homological structural analysis. The resultant structures of FtsEX with different conformations were validated using molecular dynamics (MD) simulations. Through structural analysis and biochemical functional assays, we also identified the amino acid residues that are important for the function of FtsEX. Together, our results proposed a mechanotransmission mechanism through the FtsEX's NBDs‐coupling helices (CHs)–TMDs–PLDs axis to activate the pathway for cell division.

## 2 RESULTS

### 2.1 The overall configuration of *E. coli* FtsEX

We attempted to determine the structures of wild‐type FtsEX from *E. coli*, however, the unbound/apo FtsEX protein was extremely unstable in vitro, precipitating quickly during purification. To stabilize the complex, we modified FtsEX by mutating the ATP hydrolytic residue of FtsE(163) from glutamic acid to glutamine (FtsE(E163Q)X) to retain endogenously captured ATP. The purified FtsE(E163Q)X protein was reconstituted into lipid nanodiscs, and the structure was resolved by cryo‐EM at a resolution of 2.6 Å (Figures 1D,E, S1B–D, S2, and S3A). The high resolution of the cryo‐EM map enabled the unambiguous assignment of the complex, except for the PLDs which exhibited high flexibility and therefore were built with the assistance of AlphaFold‐predicted model (Figure S3B).

The overall FtsE(E163Q)X structure adopts a compact configuration. Both the dimers of FtsE and FtsX exhibit closed‐up interfaces. Two densities of ATP molecules are situated at the interface of the NBDs between the α‐helical subdomain of one FtsE and the RecA subdomain of the other FtsE (Figure 1E). The ATP‐bound FtsE(E163Q)X structure resembles the ATP‐bound MacB structure (PDB: 5lj7), with a root mean square deviation (RMSD) of 2.55 Å over 787 residues aligned (Figures S3C and S4). FtsE contains motifs conserved for the NBDs of ABC transporters, comprising the Walker A (G35‐T43), Q loop (M83‐Q86), ABC signature motif (L138‐Q143), the Walker B (V158‐E163), the D loop (G166‐D169), and the H loop (L191‐D197) monomer (Figure 1C). FtsX contains four transmembrane helices (TM1‐4) in each monomer. Eight TMs from both FtsX units constitute the TMDs of the complex. The transmembrane helices TM1 and TM2 from both FtsX units form a central quadruple helical core extending into the periplasm, where the segment between TM1 and TM2 folds into intertwined crown‐like PLDs (Figure 1C,E). TM3 and TM4 at the periphery interact with the central core at the front and back of the interface, forming trio‐bundles (TM1–TM3–TM2’) in a front‐to‐back symmetry in contrast to the left‐to‐right symmetry of the FtsX dimer^30^ (Figure 1E). At the cytoplasmic side, the elbow helix (EH) at the N‐terminus of FtsX extends parallel to the membrane, and the CHs between TM2 and TM3 interact with FtsE in the cytoplasm (Figure 1E).

### 2.2 Allosteric communications between FtsE and FtsX

**FIGURE 2:**
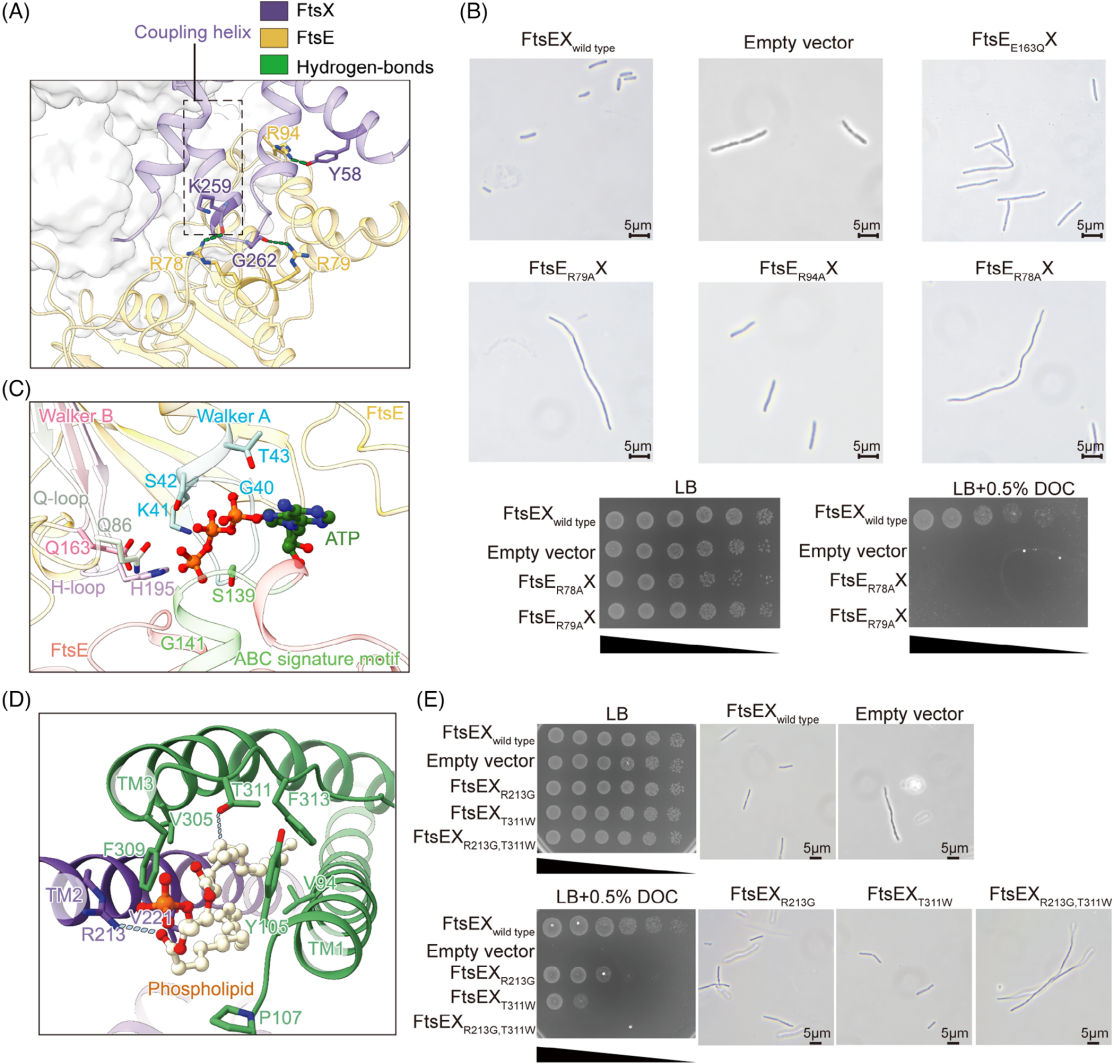
Identification of functional residues in the nucleotide‐binding domains (NBDs) and transmembrane domains (TMDs) of FtsEX. (A) The interface between FtsE (gold) and FtsX (purple), showing the coupling helix‐interacting residues. (B) Mutagenesis of the coupling residues FtsE_R78A_X and FtsE_R79A_X impeded cell growth, demonstrating filamentous morphological phenotypes and diminished cell viability. (C) The ATP‐binding pocket of the FtsE, showing interactions with the ATP molecule. The FtsE subdomains are shown in different colors. ATPs are shown as spheres. (D) The binding of a phospholipid by the trio‐bundle of FtsX, showing the interactive residues FtsEX_R213_ and FtsEX_T311_. TM1 and TM3 from one FtsX unit are presented in green and TM2 from the other FtsX is in purple. Phospholipids are shown as spheres. (E) The double mutant of the phospholipid‐interacting residues FtsEX_R213G,T311W_ impaired cell growth, demonstrating filamentous morphological phenotypes and diminished cell viability.

FtsX and FtsE form a complex through multiple intermolecular interactions (Figure S5A). Specifically, the CHs of FtsX fit into the groove at the interface of the α‐helical and RecA‐like subdomains of each FtsE unit, making hydrogen bonds between FtsX(K259) and FtsE(R78) as well as FtsX(G262) and FtsE(R79) (Figures 2A and S5A). FtsX also interacts with FtsE through the EH between FtsX(Y58) and FtsE(R94) (Figures 2A and S5A). These interactions are hypothesized to enhance the association between FtsX and FtsE, potentially preventing the dissociation of the complex during conformational changes.^31^ To confirm this, we substituted the CH‐ or EH‐interacting arginine FtsE(R78), FtsE(R79), and FtsE(R94) with alanine, and assessed their effects on protein complex stability and bacterial morphology and viability. Protein analysis of the CH‐interacting mutants FtsE(R78A)X and FtsE(R79A)X showed a reduced association between FtsE with FtsX compared to the wild type (Figure S6A), suggesting that the CHs are indeed important in mediating the association between the NBDs and TMDs. Moreover, the CH‐interacting mutants FtsE(R78A)X and FtsE(R79A)X also exhibited filamentous phenotype and impaired cell growth (Figure 2B). In contrast, the EH‐interacting mutant FtsE(R94A)X showed normal cell morphology or growth (Figures 2B and S6A). This suggests that the coupling between the NBDs and TMDs is important for the function of FtsEX, which is likely required in allosteric communications between the domains.

In the structure of FtsE(E163Q)X, two ATP molecules are interacted by FtsE through eight hydrogen bonds, including residues G40‐T43 in the Walker A, Q86 in the Q loop, S139 and G141 in the ABC signature motif, and H195 in the H loop of FtsE (Figures 2C and S5B). The binding of ATP brings two NBDs together, resulting in a closed conformation of the complex, a typical allosteric state of a type VII transporter.^17^, ^18^ E163 in the Walker B is highly conserved and involved in ATP hydrolysis.^23^, ^32^ Morphological assessment of the mutant FtsE(E163Q)X exhibited a pronounced cell division defect (Figure 2B), suggesting that ATP hydrolysis is crucial for the function of FtsEX in cell division. Previous structural studies of the lipoprotein transporter LolCDE implicate that ATP binding induces conformational changes in the complex to expel substrate and ATP hydrolysis resets the complex for substrate extraction for the next round of transport.^18^ Although FtsEX does not contain a cognate substrate, its homologous ATP‐bound conformation suggests that FtsEX likely adopts a similar allosteric mechanism in response to ATP binding and hydrolysis cycles.

### 2.3 Ligand binding stabilizes FtsEX complex

Interestingly, additional density corresponding to a pair of phospholipid molecules was found at the interface of the FtsX dimers at the periplasmic end of the TMDs (Figures 2D,E and S3B). Such an observation has not been reported in other homologous structures of FtsEX reconstituted in detergents peptidisc.^21^, ^27^, ^28^ The fitted phospholipid molecules adopt an upright orientation with the hydrophilic head oriented toward the PLDs while the hydrophobic tails are embedded within the TMDs of FtsX (Figure S3A). Notably, these phospholipid molecules are enclosed and interacted by the helical trio‐bundles (Figure 2D). The phosphate groups form hydrogen bonds with T311 on the loop between TM3 and TM4 and R213 on TM2 of the opposite FtsX. The fatty acid tails interact with the hydrophobic residues V305, F309, F313 of TM3, and V94, Y105, P107 of TM1 (Figure 2D). To test whether the presence of the phospholipid molecules is important for FtsEX function or stability, we disrupted the hydrogen bonds for phospholipid binding by substituting the polar residue T311 or R213 of FtsEX with nonpolar residues and performed functional assays using an *E. coli ftsEX* knock‐out strain. The results showed that single‐point mutant FtsEX(R213G) or FtsEX(T311W) attenuated bacterial growth, while the double‐point mutation FtsEX(R213G, T311W) completely abolished bacterial cell growth in the viability assay and showed prominent morphological changes (Figure 2E). In contrast to the rod‐shaped morphology of the wild type, the mutants displayed elongated filamentous phenotypes, implicating defective cell division. Further assessment of the mutant protein expression unveiled that the double‐point mutation caused the dissociation of FtsX and FtsE (Figure S6A). These results suggest that the presence of phospholipids may play a role in stabilizing the FtsEX complex.

Compared to unbound FtsEX, ligand binding seems to improve the stability of the FtsEX complex. To emphasize this, we performed a fluorescence resonance energy transfer (FRET) assay to assess the association between FtsE and FtsX in wild‐type versus the ATP‐bound FtsE(E163Q)X. We fused the enhanced cyan fluorescent protein (ECFP) to FtsX as the donor chromophore and the enhanced yellow fluorescent protein (EYFP) to FtsE as the acceptor chromophore. The association of FtsX–ECFP and FtsE–EYFP is reflected by the fluorescent emission shift from the donor to the acceptor when the two fluorophores are in proximity allowing energy transfer. The results showed that the purified wild‐type protein emitted at 470 nm (emission wavelength for the donor ECFP), while the mutant protein emitted at 520 nm (emission wavelength for the acceptor EYFP; Figure S6B). This suggests that the purified unbound FtsEX will likely dissociate, while the ATP‐bound FtsEX is stable as an intact complex. This explains why determining the true apo state of FtsEX has been challenging.

### 2.4 Allosteric characterization of FtsEX–EnvC

Prior studies have demonstrated that PG hydrolysis requires FtsEX‐mediated EnvC activation,^23^ and the conformational changes of EnvC are coupled with that of FtsEX.^33^ However, the details about how FtsX interacts with EnvC and regulates the downstream AmiB amidase induced by ATP remain unexplored. To investigate the conformational changes within the FtsEX complex driven by ATP binding and hydrolysis cycles and to explore how signals are transmitted from FtsEX to EnvC for the subsequent activation of the amidase AmiB, we conducted structural analysis of FtsEX and FtsEX–EnvC complexes in both the apo and ATP‐bound states.

**FIGURE 3:**
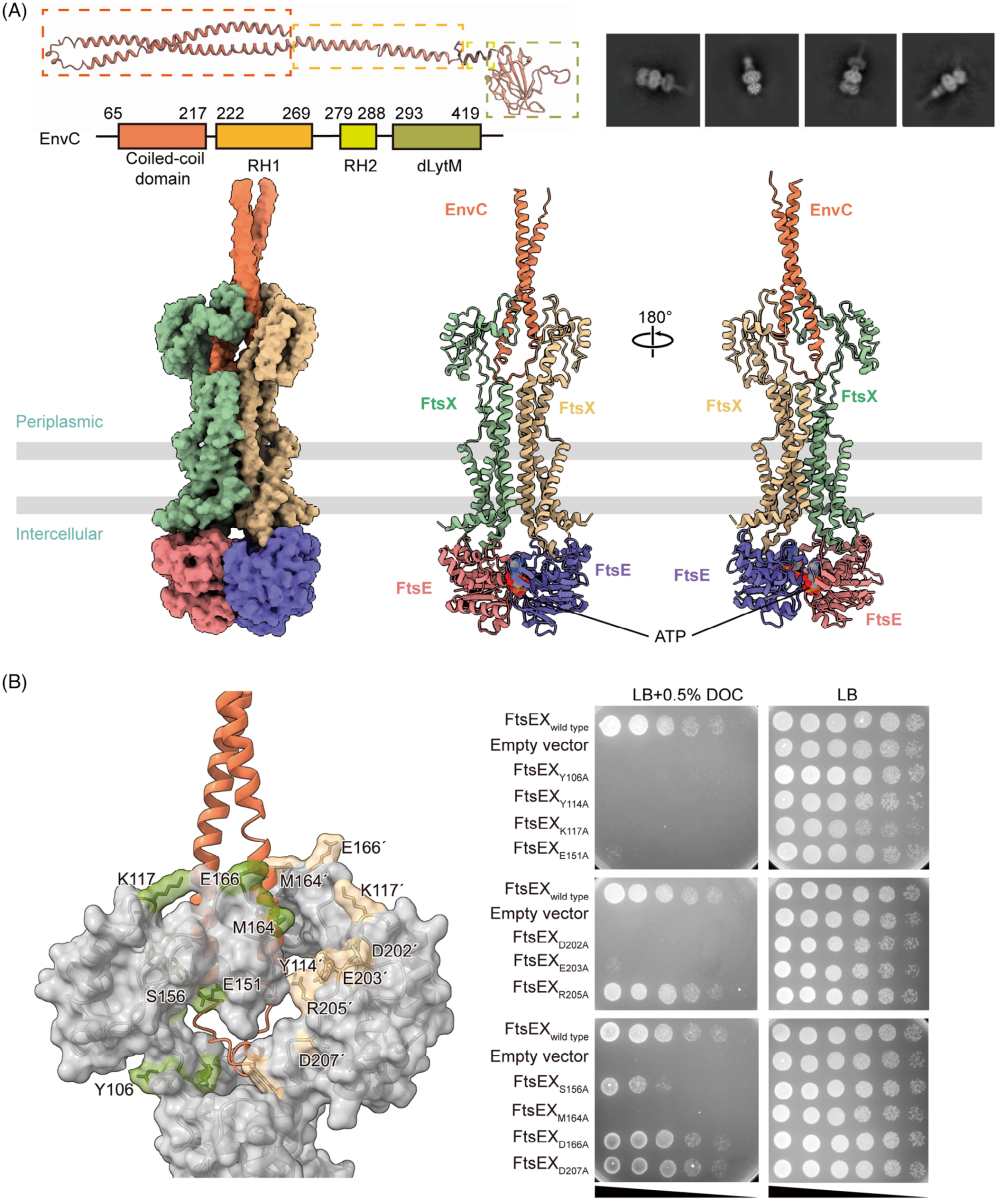
Structural characterization of FtsE(E163Q)X‐EnvC by cryogenic electron microscopy (cryo‐EM). (A) The AlphaFold‐predicted structure and topology of EnvC (left panel). The cryo‐EM particle images of FtsE(E163Q)X‐EnvC complex (right panel). The cryo‐EM density map and structure of the FtsE(E163Q)X‐EnvC complex (lower panel). (B) The zoomed surface of the periplasmic loop domains (PLDs) in FtsE(E163Q)X‐EnvC, showing interface residues associated with the N‐terminus of EnvC (left panel). Mutagenesis of the interface residues of FtsX Y106A, Y114A, K117A, E151A, D202A, E203A, and M164A showed growth defects.

**FIGURE 4:**
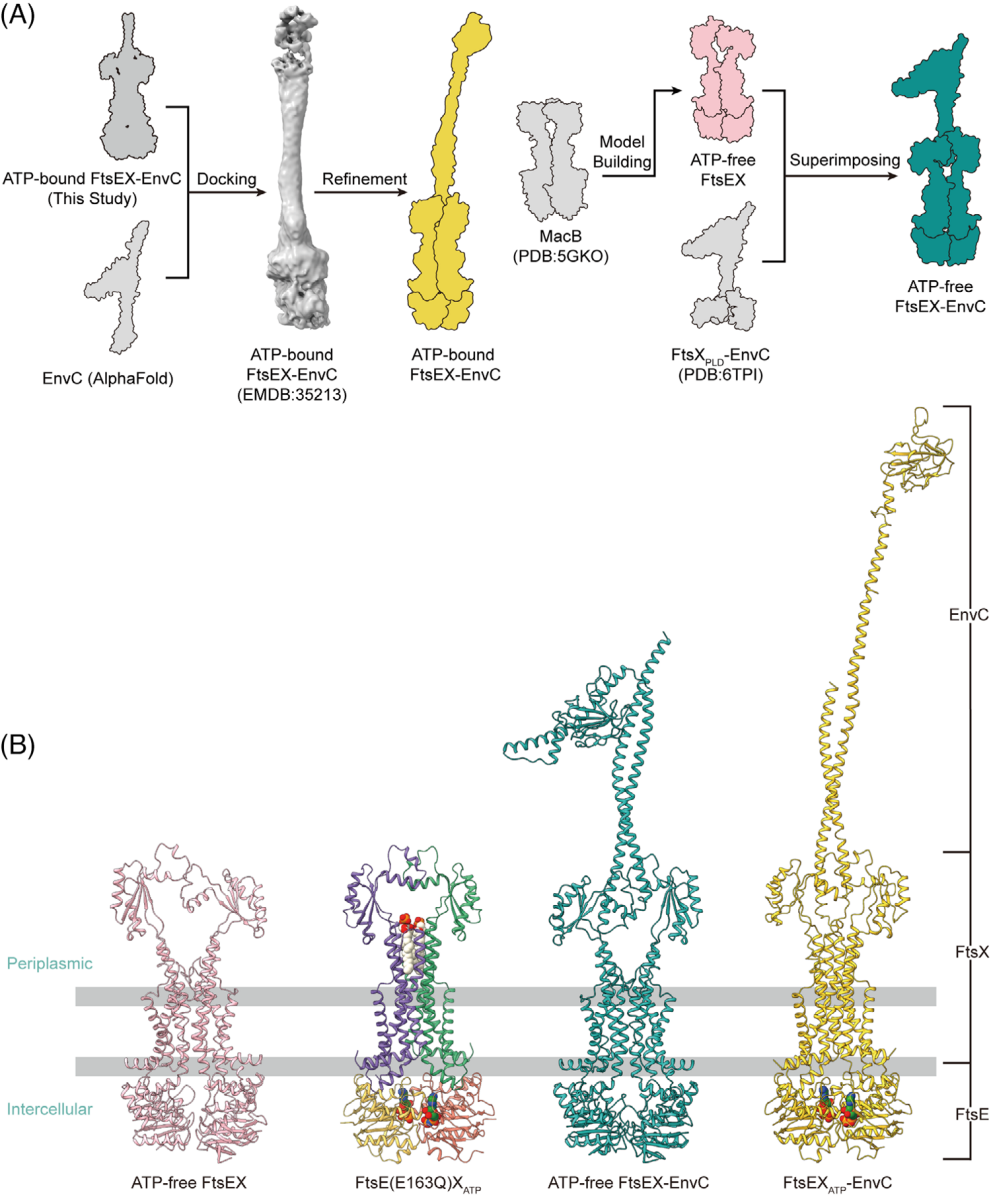
Homology modeling of the apo FtsEX and apo FtsEX–EnvC structures. (A) Flowchart detailing the homology modeling process for ATP‐free FtsEX, ATP‐free FtsEX–EnvC, and ATP‐bound FtsEX–EnvC (modeled for the C‐terminus of EnvC). (B) The structures of ATP‐free FtsEX (modeled), ATP‐bound FtsE(E163Q), ATP‐free FtsEX–EnvC (modeled), and ATP‐bound FtsE(E163Q)X‐EnvC, from left to right.

We first determined the structure of FtsEX–EnvC through cryo‐EM. FtsE(E163Q)X and EnvC were coexpressed from *E. coli* and purified as a complex, and the cryo‐EM structure of FtsE(E163Q)X‐EnvC was resolved at an overall resolution of 4.25 Å (Figures 3A and S7). Though the resolution at the periplasmic region of the structure is relatively low, there is a clear columnar density on top of the PLDs of the FtsEX, resembling the N‐terminal helical structure of EnvC (Figure 3A). EnvC comprises four domains: the N‐terminal coiled‐coil (CC) domain, regulatory helix 1 (RH1), RH2, and the C‐terminal dLytM domain^21^, ^38^ (Figure 3A). In the structures of FtsE(E163Q)X‐EnvC, the N‐terminus of EnvC features an antiparallel coiled coils hairpin, inserted into the interface of the PLD of FtsX (Figure 3A). The clamp‐like structures of FtsX's PLDs grasp the coiled‐coil structure of EnvC making multiple contacts (Figure 3B). Mutations of the EnvC surrounding residues in the PLDs of FtsX abrogated cell growth (Figure 3B), implicating their involvement in coupling with EnvC. As the tenuous structure of EnvC exhibits high flexibility, the far‐end C‐terminus of EnvC in our structure of FtsE(E163Q)X‐EnvC was not resolved. Searching through the EM data bank, there is only one deposited FtsEX–EnvC cryo‐EM map showing an extended EnvC density with an upright gesture at the far end at a resolution of 19.7 Å (EMDB: 35213). Based on our determined N‐terminal structure of EnvC, we completed the model of EnvC using the upright extended map of EnvC (EMDB: 35213) as a guide to position the C‐terminal domain of AlphaFold‐predicted EnvC for the ATP‐activated conformation (Figure 4A,B).

We also acquired the apo FtsEX and apo FtsEX–EnvC structure by homologous modeling as the apo state of FtsEX protein is unstable in vitro (Figure 4A). MacB is the most thoroughly characterized type VII ABC transporter protein complex.^17^ The ATP‐bound MacB exhibits a high structural homology to ATP‐bound FtsEX with an RMSD of 2.55 Å when superimposed (Figure S3C). Therefore, we constructed the model of the apo FtsEX based on the apo structure of MacB (PDB ID: 5GKO) using the SWISS‐MODEL server^34^, ^35^, ^36^ (Figure 4A). Consequently, we also modeled the apo FtsEX–EnvC structures by manually docking the apo FtsEX structure into the crystal structure of FtsX_PLD_–EnvC (PDB ID: 6TPI) using Coot^21^ by overlapping the PLDs of the structures (Figure 4A).

### 2.5 MD simulation of the FtsEX–EnvC complex

**FIGURE 5:**
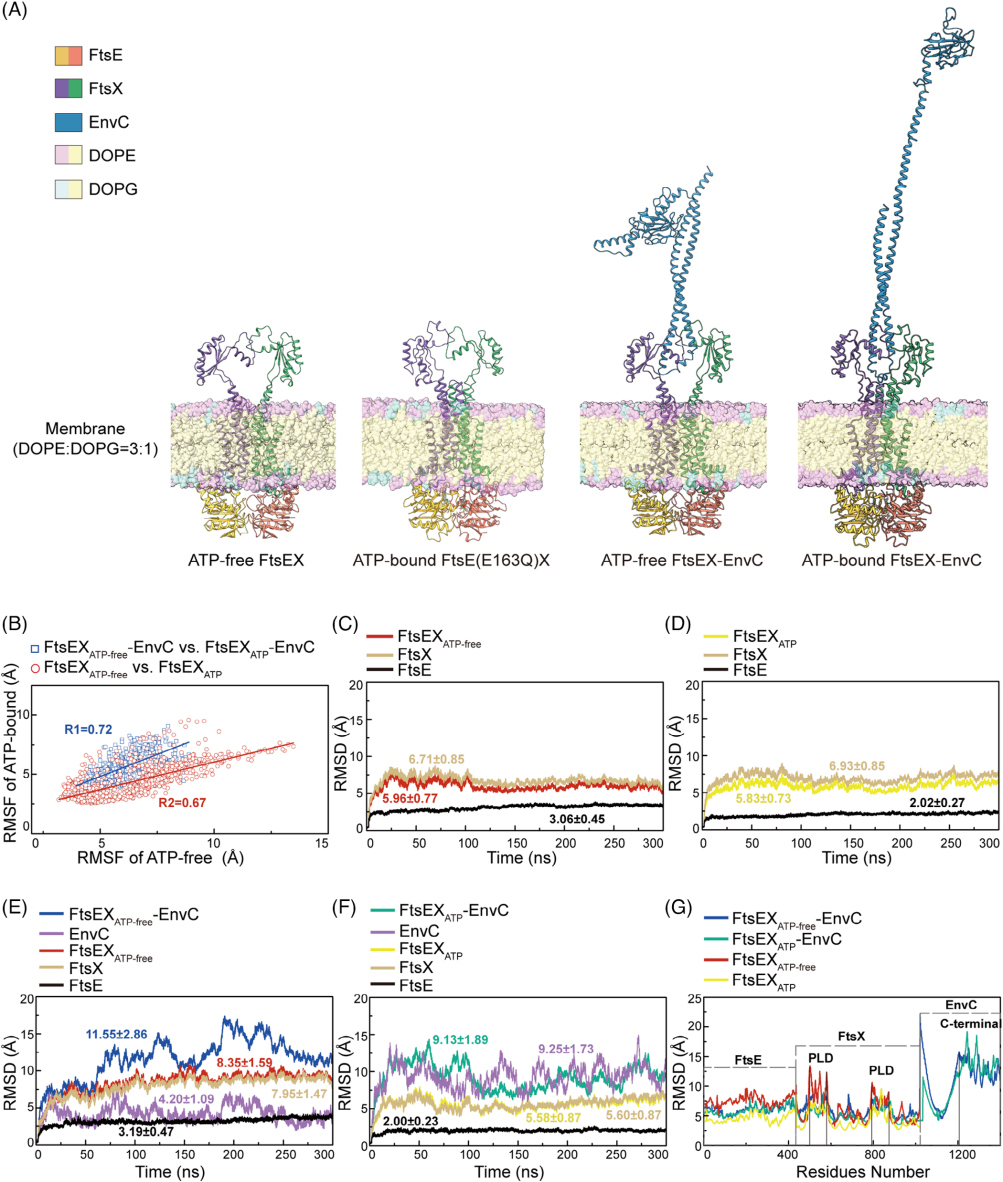
Molecular dynamic (MD) simulations of FtsEX and FtsEX–EnvC structures. (A) The modeled structures of FtsEX, ATP‐bound FtsEX, FtsEX–EnvC, and ATP‐bound FtsEX–EnvC embedded in a modeled phospholipid membrane. (B) Linear fitting of root mean square fluctuation (RMSF) from 300 ns MD simulations of FtsEX and FtsEX–EnvC performed both with and without ATP. (C–F) Root mean square deviation (RMSD) analyses were conducted for each component in ATP‐free FtsEX (C), ATP‐bound FtsEX (D), ATP‐free FtsEX–EnvC (E), and ATP‐bound FtsEX–EnvC (F), respectively. (G) RMSF analyses for ATP‐free FtsEX, ATP‐bound FtsEX, ATP‐free FtsEX–EnvC, and ATP‐bound FtsEX–EnvC.

To speculate the MD of FtsEX–EnvC, simulations were performed by reconstituting the structures of FtsEX, ATP‐bound FtsEX, FtsEX–EnvC and ATP‐bound FtsEX–EnvC in a lipid environment composed of a 3:1 ratio of phospholipids DOPE and DOPG using PACKMOL‐Memgen^37^ (Figure 5A). The simulations ran for 300 ns, with the models embedded within lipid bilayers (Figure 5A). We measured RMSD for each structure in the simulated condition and performed linear fitting to the root mean square fluctuation (RMSF) data for conformational variations between the ATP‐free and ATP‐bound states (Figure 5B). The low correlation coefficient between the ATP‐free and ATP‐bound structures suggests that the dynamics of the protein complex change in response to ATP binding. In particular, the MD simulations of the FtsEX structure show a lower RMSD for the FtsE (3.06 ± 0.45 Å in ATP‐free state and 2.02 ± 0.27 Å in ATP‐binding state) than for FtsX (6.71 ± 0.85 Å in ATP‐free state and 6.93 ± 0.85 Å in ATP‐binding state), and the RMSD profile trend for the overall structure matches that of the FtsX (Figure 5C,D). These suggest that FtsX is more flexible than FtsE and the conformational fluctuations of the complex are mainly attributed to FtsX. The RMSD value for FtsE is lower in the ATP‐bound state compared to the ATP‐free state (2.02 ± 0.27 Å vs. 3.06 ± 0.45 Å, respectively; Figure 5C,D), indicating an enhanced conformational stability of FtsEX in response to the ATP‐induced closure of the NBDs. This phenomenon was also observed in the homologous proteins MacB and LolCDE, for which the ATP‐induced NBDs closed protein complex exhibits the greatest stability in vitro.^11^, ^18^

Simulations of the FtsEX–EnvC structures showed lower RMSD values for the overall structure in the presence of ATP relative to its absence (9.13 ± 1.89 Å vs. 11.55 ± 2.86 Å, respectively; Figure 5E,F). Intriguingly, under identical conditions, the RMSD for EnvC within the FtsEX–EnvC complex was increased upon ATP binding (4.20 ± 1.09 Å vs. 9.25 ± 1.73 Å), suggesting a tendency of conformational changes in EnvC in the ATP‐bound structure. RMSF analysis revealed higher fluctuations in the PLD of FtsX and the C‐terminal region of EnvC, indicating higher conformational flexibility in these regions (Figure 5G). Furthermore, free energy landscape (FEL) analysis and conformational cluster analysis of the PLDs suggest that in both FtsEX and FtsEX–EnvC structures, the PLDs exhibit higher conformational variation in the absence of ATP (Figure S8). This implies that the PLDs are more stable in the ATP‐bound conformation, which is probably attributed to the increased interactions at the interface with EnvC in response to the NBDs‐induced conformational changes in the PLDs and TMDs. Nevertheless, the RMSF fittings for these structures generally match (Figure 5G), suggesting that the models built are consistent with the cryo‐EM structures of FtsEX under the condition of lipid environment.

### 2.6 Mechanotransmission of FtsEX to EnvC

Studies on MacB and LolCDE have elucidated a mechanotransmission mechanism for propagating conformational changes across the membrane,^11^, ^18^ which advances our comprehension of mechanical principles in the Type VII ABC transporters. The ATP binding to FtsEX induces closed NBDs, indicating that the dimerization of FtsE is requisite for FtsEX function. To investigate the conformational changes associated with FtsEX, we compared the FtsEX–EnvC structures in the presence or absence of ATP.

**FIGURE 6:**
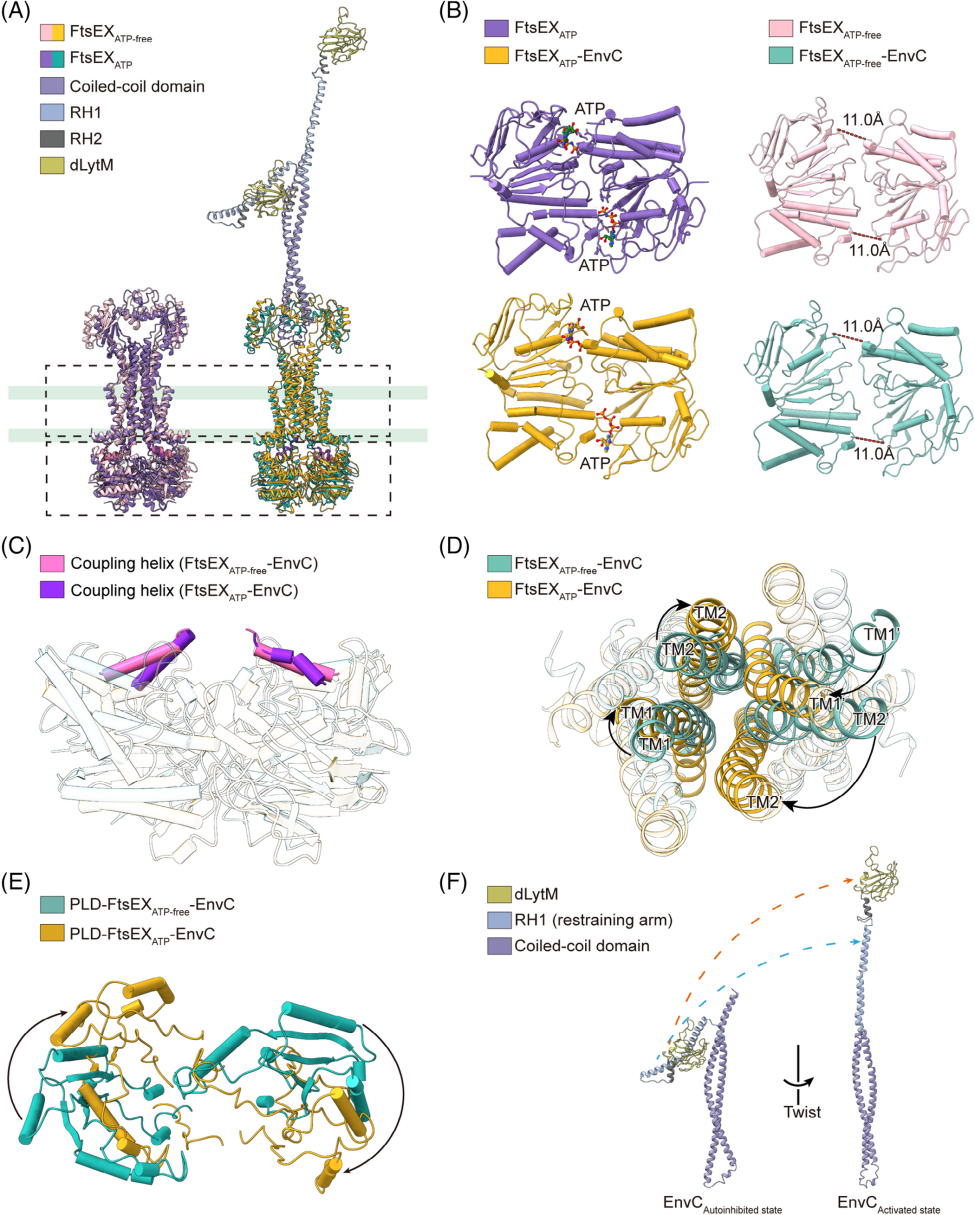
Conformational changes of FtsEX‐ and EnvC‐induced ATP binding. (A) Autoinhibited and activated state of EnvC in the FtsEX–EnvC complex. (B) Structural comparisons showing unchanged nucleotide‐binding domain (NBD) interface (top view) with or without the binding of EnvC, either in the presence of ATP (purple and yellow) or absence of ATP (pink and green). (C) Structural superimposition of the NBDs of FtsEX–EnvC (side view) with and without ATP, showing the conformational changes of the coupling helices (colored in bright) between the two states. (D–F) Structural superimpositions show conformational changes of transmembrane helices (top view) (D), the periplasmic loop domains (PLDs; top view) (E), and EnvC (F) between the ATP‐bound and free states.

Previous studies have shown that EnvC can exist in two states, the active and inactive, characterized by whether the conformation of EnvC's C‐terminal RHs and dLytM domains allows the binding and activating of the amidase AmiB.^6^, ^25^ The inactive state is signified by the self‐inhibition of EnvC, a state primarily regulated by its RH1 domain, often referred to as the restraining arm.^33^ In the self‐inhibited conformation of EnvC, the C‐terminal RHs and the dLytM domains fold back onto the N‐terminal coiled‐coil, preventing the dLytM domain from binding to AmiB.^33^ The restraining arm is sprung into a relatively upright conformation in the active conformation, which fully releases the dLytM domain to reach the PG layer (Figure 6A). Superimpositions of the structures reveal remarkable conformational changes in the TMDs and PLDs that interact with the EnvC, evidenced by an RMSD of 4.078 Å over 834 aligned atoms between FtsEX and ATP‐bound FtsEX, and an RMSD of 17.708 Å over 1036 aligned atoms between FtsEX–EnvC and ATP‐bound FtsEX–EnvC (Figure 6B,C). The closure of the NBDs in the ATP‐bound FtsEX structure has induced an inward shift of the CHs in TMD (Figure 6D) and a clockwise rotary shift in the TMDs from the top view (Figure 6E). In the ATP‐free structure, the central core of FtsX becomes separated, forming a V‐shaped cavity at the interface (Figure 4B). TM2 in one FtsX monomer is away from the TM1 and TM3 of the opposite FtsX, which is no longer able to form a trio‐bundle (Figure 6E). The resulting PLDs between the structures also exhibit rotational conformational changes (Figure 6F), which change the conformation at the interface between FtsX and EnvC. In the ATP‐bound structure, additional hydrogen bonds establish a stable and compact conformation at the interface between FtsX and EnvC (Figure S9A). As a result, the coiled springs of EnvC are wrung from the N‐terminus by the rotational closure of the FtsX, which induces the transition of the restraining arm from the inactive to the active state to release the C‐terminal AmiB‐binding dLytM domain (Figure 6A,B).

## 3 DISCUSSION

**FIGURE 7:**
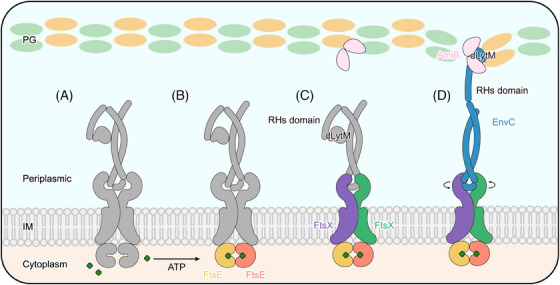
Schematic diagram of the proposed mechanotransducing mechanism of FtsEX. (A) In the ATP‐free state, FtsEX adopts a relaxed conformation. (B) Upon ATP binding, FtsE (nucleotide‐binding domain [NBD]) dimerizes. (C) FtsE dimerization results in the conformational changes in transmembrane domains (TMDs) and periplasmic loop domains (PLDs). (D) Subsequently, the rotatory torsion of the PLDs induces EnvC to open the RHs domain and expose the dLytM domain. This cascade of conformational changes in FtsEX induces EnvC to transit from a self‐inhibited to an active state, ultimately activating the downstream protein AmiB to hydrolyze peptidoglycan (PG).

In this study, we resolved the high‐resolution cryo‐EM structures of the ATP‐bound FtsE(E163Q)X and FtsE(E163Q)X‐EnvC from *E. coli*. Interestingly, we observed the binding of phospholipids in the structure acquired in lipid nanodiscs, which has not been reported in previous studies. We speculate that the phospholipids stabilize the functional conformation of FtsEX in response to ATP binding, thereby mediating the activation twist in EnvC. Indeed, site‐directed mutagenesis of the phospholipid‐binding residues caused bacterial cell lethality and molecular dissociation of FtsX and FtsE. The localization of these residues in the periplasm makes them a potential target for antimicrobial treatment. Comparison between our experimental and modeled structures reveals the conformational dynamics of the FtsEX–EnvC complex. Initially, FtsEX adopts a relaxed conformation in the ATP‐free state, regardless of whether EnvC is bound. Upon ATP binding, FtsE (NBDs) closes, converting the complex to a more compact conformation. The closure of the FtsE dimer causes the CHs of FtsX to approach each other, consequently inducing the TMs to draw closer and the PLDs to undergo a rotational conformational change. This rotation at the interface between FtsX and EnvC twists the coiled‐coil into a tightened conformation such that erecting the effector domain of EnvC into an activated conformation (Figure 7). Together, our results propose an ATP‐driven mechanotransduction mechanism, transmitted along the NBDs–CHs–TMDs–PLDs axis.

Our proposed mechanism of FtsEX is similar to that of the type VII ABC transporter LolCDE. Our team, along with Sharma et al. and Bei et al., reported the cryo‐EM structures of LolCDE in both ATP‐free and ATP‐bound states from the same species (*E. coli*), and observed that ATP binding induces the closure of the NBDs of LolCDE, which in turn triggers a conformational change in the TMD^18^, ^39^, ^40^ (Figure S10). This suggests that the mechanotransduction mechanism is conserved in Type VII ABC transporters. In addition to our study, Xu et al. conducted a structural analysis of FtsEX–EnvC from *Pseudomonas aeruginosa* ^21^ (Figure S11). Their findings suggested a similar mechanism, in which the mechanical transmission by FtsEX induces conformational changes in EnvC, ultimately leading to the activation of AmiB. This research significantly advanced our understanding of the operational dynamics within the FtsEX–EnvC system.^41^ Recently, Hao et al. reported the cryo‐EM structure of *Vibrio cholerae* FtsEX–EnvC bound to ADP, revealing that FtsX undergoes a more pronounced torsional shift, leading to an almost perpendicular conformational transition in EnvC.^27^ Intriguingly, Li et al. recently investigated the complex structure of *Mycobacterium tuberculosis* FtsEX with RipC (Rv2190c) serving both adaptor and amidase activities and observed a similar mechanotransmission from cytosolic FtsE to the extracellular activation of RipC upon ATP binding^28^ (Figure S11). These studies suggest a conserved mechanotransmission mechanism of FtsEX across species. Previous studies have demonstrated that once EnvC is activated, it further regulates the downstream protein AmiB.^42^ AmiB acts as a PG amidase, which cleaves the cross‐linkages between the PG chains.^6^, ^43^ The study by Cook et al. preliminarily showed that EnvC induces significant conformational changes in AmiB, thus exposing the active site and transitioning from a self‐inhibitory state to an active state.^33^ Despite the studies of FtsEX from several species, none of these have captured the actual apo state of FtsEX due to the instability of the unbound form of this protein as assessed in the study by FRET. Therefore, to unveil the molecular details between protein components at an atomic level, further optimization is required.

In summary, the ATP‐induced mechanotransduction by FtsEX ensures the regulated hydrolysis of PG in *E. coli* during the cell division process. It collaborates with other members of the divisome, playing a pivotal role in normal bacterial proliferation. This perspective provides new insights for drug development targeting antibacterial therapies.

## 4 METHODS AND MATERIALS

### 4.1 Overexpression and purification of FtsEX and FtsEX–EnvC

The genes encoding *ftsE* and *ftsX* were amplified from *E. coli* K12 genomic DNA using polymerase chain reaction (PCR) and inserted into pET‐28a plasmid by homologous recombination. An N‐terminal 8 × His tag and SUMO fusion protein were inserted into *ftsE*. The resulting pET‐28a‐8 × His‐SUMO‐FtsEX vector underwent the introduction of a FtsE (E163Q) mutation by site mutagenesis PCR and then was transformed into *E. coli* C43 (DE3) for expression.

*E. coli* C43(DE3) cells containing FtsE(E163Q)X were cultured at 37°C for 2 h after the addition of 50 µg/mL kanamycin and then induced by the addition of 0.2 mM IPTG at an OD600 of 0.8 for 12 h at 20°C. The induced cells were collected by centrifugation and then resuspended in buffer A (50 mM 4‐(2‐hydroxyethyl)‐1‐piperazineethanesulfonic acid (HEPES), 300 mM NaCl, 10% glycerol, and 1 mM ATP, pH 7.8). The cells were lysed by a high‐pressure homogenizer, and the lysate were ultracentrifuged at 100,000 × *g* at 4°C for 1 h. Buffer A containing 1% (w/v) *n*‐dodecyl‐β‐d‐maltoside (DDM, Anatrace) was used to extract proteins for 30 min at room temperature, followed by ultracentrifugation at 100,000 × *g* at 4°C for 1 h. The supernatant was bound to a HisTrap HP column (5 mL, GE HealthCare). Bound proteins were eluted using buffer A containing 300 mM imidazole and 0.02% (w/v) glyco‐diosgenin (GDN, Anatrace). The eluted sample was concentrated and applied to a size exclusion chromatography column (Superdex 200 Increase 10/300 GL column, GE Healthcare) in buffer B (20 mM HEPES, pH 7.5, 150 mM NaCl, 1 mM ATP) containing 0.02% (w/v) GDN.

For optimized expression, truncated EnvC (aa35–aa419) fused with N‐terminal SUMO and C‐terminal Strep‐II tag were cloned into pET‐28a plasmid. The resulting pET‐28a‐SUMO‐EnvC(35‐419)‐Strep vector was transformed into *E. coli* C43 (DE3) for expression. Purified SUMO‐EnvC(35‐419) from the supernatant of the cell lysate was enzymatically treated to remove the SUMO fusion and incubated with the purified FtsE (E163Q)X protein at room temperature for 1 h in a molecular ratio 10:1. The FtsE (E163Q)X‐EnvC complex was again pulled down through nickel affinity chromatography.

### 4.2 Nanodisc reconstitution of FtsE(E163Q)X

Nanodisc reconstitution of FtsE(E163Q)X was performed according to the method of Denisov et al.^44^ Briefly, chloroform‐solubilized *E. coli* polar extract was blown dry using nitrogen, and then the extract was dissolved using buffer B containing sodium cholate to dissolve and sonicate until clarified. The purified FtsE_E163Q_X was mixed with MSP1D1 and *E. coli* polar extract solution at molar ratio of 1:2:200 and incubated at 4°C for 1 h. Then the detergent was removed using BioBeads SM‐2 (Bio‐Rad). Then, use the Superdex 200 Increase 10/300 column (GE Healthcare) to purify. Protein fractions with the highest purity were pooled and concentrated to 4 mg/mL.

### 4.3 Construction of the ∆ftsEX *E. coli* MG1655 knock‐out strain

The *ftsEX* gene in the *E. coli* MG1655 strain was disrupted using λ‐Red recombination and was substituted with a chloramphenicol‐resistant gene. Positive colonies were verified through PCR and gene sequencing.

### 4.4 Cell viability assays

To observe whether mutations affect bacterial growth, we conducted in vivo functional experiments. Mutations were transformed into Δ*ftsEX* strain and cultured overnight on Lysogeny broth (LB) agar plates at 37°C. Single colonies were then inoculated into 6 mL LB medium and cultured at 37°C for 10 h. A vector containing the wild‐type *ftsEX* gene was transformed into the Δ*ftsEX* strain as a positive control, while an empty vector lacking the *ftsEX* gene served as a negative control. After culturing, cells were collected and diluted in LB medium to OD600 of 1.0, then followed by 10‐fold serial dilutions for functional assays. The dilutions ranged from 10^−1^ to 10^−6^, and the diluted cells were dripped onto LB agar plates with or without 0.5% sodium deoxycholate (DOC, Aladdin) and incubated overnight at 37°C to record cell growth.

### 4.5 Bacterial morphology assay

To investigate the impact of mutations on bacterial division, the constructed FtsEX wild‐type vector was mutated and transformed into the *∆ftsEX* strain. The transformants were cultured on an LB plate at 37°C overnight. A single colony was inoculated into 3 mL LB broth with antibiotics and cultured overnight at 37°C. The overnight culture was then diluted 100‐fold in low‐salt LB, induced with 0.2 mM IPTG, and cultured at 42°C for 3 h. Bacterial morphology was observed using a microscope (Zeiss).

### 4.6 Fluorescence resonance energy transfer assay

Fragments for EYFP–FtsE and FtsX–ECFP were obtained by PCR and cloned into plasmid pET28a with eight histidine tags (8 × His) to produce plasmids pet28a‐eyfp‐ftsex‐ecfp and pET28a‐eyfp‐ftse(E161Q)x‐ecfp. The control plasmids pet28a‐eyfp‐ecfp, pet28a‐eyfp, and pet28a‐ecfp were also constructed. All plasmids were transformed into *E. coli* C43 (DE3; Novagen) for protein expression and the proteins were purified as described above and loaded into a black 96‐well plate. The excitation and emission wavelengths for ECFP, EYFP, ECFP–EYFP, EYFP–FtsEX—ECFP, and EYFP–FtsE(E161Q)X–ECFP were monitored. The emission signals for ECFP (donor), EYFP (acceptor), and ECFP–EYFP were used as controls. All samples are tested by Cytation3 Elisa Analyzer. The excitation light wavelength for ECFP is 458 nm, and the emission light detection range is 450 nm to 600 nm.

### 4.7 Construction of the computational models

Four membrane‐based atomistic models were generated for MD simulations. The ATP‐bound model of FtsEX was determined using cryo‐EM in our study. The ATP‐free model of FtsEX was constructed based on crystal data as well as the ATP‐free state of the homologous ABC transporter MacB (PDB ID: 5GKO) using the SWISS‐MODEL server.^34^, ^35^, ^36^ The initial model of EnvC originated from a crystal structure of the FtsX_PLD_ in complex with autoinhibited EnvC (PDB ID: 6TPI).^33^ The ATP‐free model of the FtsEX–EnvC complex was generated by superposing the PLD of FtsX_PLD_–EnvC and ATP‐free FtsEX. To construct the ATP‐bound FtsEX–EnvC model, AlphaFold‐predicted EnvC structure was manually docked into the cryo‐EM map (EMDB‐35213)^21^ for the C‐terminus and the resulting structure is modeled into our FtsEX–EnvC cryo‐EM map to complete the structure. All structures were subjected to real space refinement in Phenix.^41^, ^45^, ^46^

### 4.8 Membrane molecular dynamics simulation

All systems were embedded in a lipid bilayer with a composition of DOPE: DOPG as 3:1, which is a simplified representation of such a system for *E. coli* using the Memgen sector of PACKMOL as the packing engine for the MD simulation. Structural figures were created using PyMOL.^47^ MD simulations were carried out using the ff19SB force field and Amber 19 package.^48^, ^49^ The SHAKE algorithm was used to constrain the bonds containing hydrogens to equilibrium length. Simulations were conducted in the NPT ensemble with *P* = 1 bar, using the Berendsen barostat.^50^ The simulation temperature was set at 300 K and the systems were solvated with 150 mM NaCl in the TIP3P water model where solutes were placed in the octahedral box with a boundary of 15.0 Å.^51^ All systems were initially subjected to 5000 steps of steepest descent optimization followed by another 5000 steps of conjugate gradient minimization of the potential energy. Subsequent NPT simulations were performed for 300 ns with a time step of 2.0 fs, which was sufficient for the simulation systems to reach equilibrium.

### 4.9 Cryo‐EM sample preparation and data acquisition

To prepare samples for cryo‐EM structural analysis, 3 µL of the purified FtsE(E163Q)X or FtsE(E163Q)X‐EnvC protein (concentration approximately 3.0 mg/mL) was carefully applied to glow‐discharged Quantifoil holey‐carbon grids (R1.2/1.3, 300 mesh, copper). These sample‐loaded grids were briefly blotted for 4 s and plunged into liquid ethane using a Vitrobot Mark IV (Thermo Fisher) operated at 100% humidity. The samples were imaged using a Titan Krios electron microscope (Thermo Fisher) equipped with a K2 Summit direct electron‐counting camera (Gatan). All cryo‐EM images were acquired in counting mode at a nominal magnification of 29,000×, corresponding to a calibrated physical pixel size of 1.014 Å. Each image was captured with an exposure time of 8 s, and dose fractionation was conducted at a dose rate of approximately 8 counts per pixel per second.

### 4.10 Image processing

To obtain the FtsE(E163Q)X structure, beam‐induced motion correction was carried out using MotionCor2 to align the image stacks, resulting in dose‐weighted micrographs with a pixel size of 1.014 Å.^52^ The Contrast transfer function (CTF) parameters were estimated by Gctf and particle picking was performed using Gautomatch.^53^ 2D classification, ab‐initial model, 3D classification and particle polishing were conducted in RELION, and nonuniform refinement was done using CryoSPARC.^54^, ^55^ The 2.6 Å resolution map was obtained utilizing a B‐factor of −94 Å^2^. A detailed account of the data processing workflow can be found in Supporting Information Figure 2. The overall resolution was determined based on the gold‐standard Fourier shell correlation (FSC) = 0.143.

To resolve the FtsE(E163Q)X‐EnvC structure, beam‐induced motion correction was carried out using MotionCor2 to align the image stacks, with dose‐weighted micrographs with a pixel size of 0.84 Å.^52^ The CTF parameters were estimated by Gctf and particle picking was performed using Gautomatch.^53^ 2D classification, ab‐initial model, 3D classification and particle polishing were conducted in RELION, and nonuniform refinement was done using CryoSPARC.^54^, ^55^ The 4.25 Å resolution map was obtained utilizing a B‐factor of −141.7 Å^2^. A detailed account of the data processing workflow can be found in Supporting Information Figure 7. The overall resolution was determined based on the gold‐standard FSC = 0.143.

### 4.11 Model building and refinement

The cryo‐EM map of FtsE(E163Q) X bound ATP was obtained at a resolution of 2.6 Å, which allowed the initiation of model construction using Coot.^56^ The model was then refined iteratively using Phenix and Coot.^46^ Model validation was performed using Phenix (Table S2). Figures were generated using PyMol, ChimeraX, and Chimera.^57^

## AUTHOR CONTRIBUTIONS

Xiaodi Tang, Jianping Hu, and Shenghai Chang conceived and supervised the project. Yujiao Chen, Changbin Zhang, and Qinghua Luo constructed, expressed, and purified the proteins. Yuejia Chen, Xin Wang, and Yatian Chen conducted mutagenesis, cell viability, morphology, and FRET assays. Du Guo and Jianping Hu carried out molecular dynamic simulations and prepared MD data. Shenghai Chang and Zhibo Zhang collected and processed electron microscopy data, and built and refined atomic models. Yuejia Chen, Xin Wang, Changbin Zhang, Yujiao Chen, Lili Yang, Qinghua Luo, Jianping Hu, Tian Hong, and Zhengyu Zhang performed structural modeling using AlphaFold2 and analyzed the structures. Xiaodi Tang, Haohao Dong, Yuejia Chen, Du Guo, Changbin Zhang, and Zhengyu Zhang prepared the results and wrote the manuscript, with revisions from all authors. All authors have read and approved the final manuscript.

## CONFLICT OF INTEREST STATEMENT

The authors declare no conflicts of interest.

## Supporting information

Supporting Information

## Data Availability

The data that support the findings of this study are openly available in PDB at https://rcsb.org, EMDB at https://www.ebi.ac.uk/emdb/, reference number 8YMC, EMD‐39394.
