# Supplementary material for: Structural insights into the mechanotransducing mechanism of FtsEX in cell division
Source: MedComm (2020). 2024 Oct 20;5(11):e688. doi: 10.1002/mco2.688 (PMC11491553; doi:10.1002/mco2.688)
Supplement: Supplementary file 1 — Supporting Information [file MCO2-5-e688-s001.pdf]

Structural insights into the mechanotransducing mechanism of FtsEX in cell division

Yuejia Chen[1#], Du Guo[1#], Xin Wang[1,2#], Changbin Zhang[1#], Yatian, Chen[3], Qinghua Luo[1,2], Yujiao Chen[1], Lili Yang[1], Zhibo Zhang[1], Tian Hong[1], Zhengyu Zhang[3], Haohao Dong[1,2*], Shenghai Chang[4*], Jianping Hu[5*,] Xiaodi Tang[1*]

[1]Department of Laboratory Medicine, State Key Laboratory of Biotherapy, Institute for Breast Health Medicine, National Clinical Research Center for Geriatrics, West China Hospital, Sichuan University, Chengdu, China.

[2]Frontiers Medical Center, Tianfu Jincheng Laboratory, West China Hospital, Sichuan University, Chengdu, China.

[3]Department of Clinical Laboratory, Zhongnan Hospital of Wuhan University, School of Pharmaceutical Sciences, Wuhan University, Wuhan, China.

[4]Center of Cryo-Electron Microscopy, Zhejiang University, Hangzhou, Zhejiang, China.

[5]Key Laboratory of Medicinal and Edible Plants Resources Development of Sichuan Education Department, College of Pharmacy and Biological Engineering, Sichuan Industrial Institute of Antibiotics, Chengdu University, Chengdu, China.

*Correspondence: Shenghai Chang (changshenghai@zju.edu.cn), Jianping Hu (hjpcdu@163.com) or Xiaodi Tang (tangxiaodi@scu.edu.cn)

Supporting Information

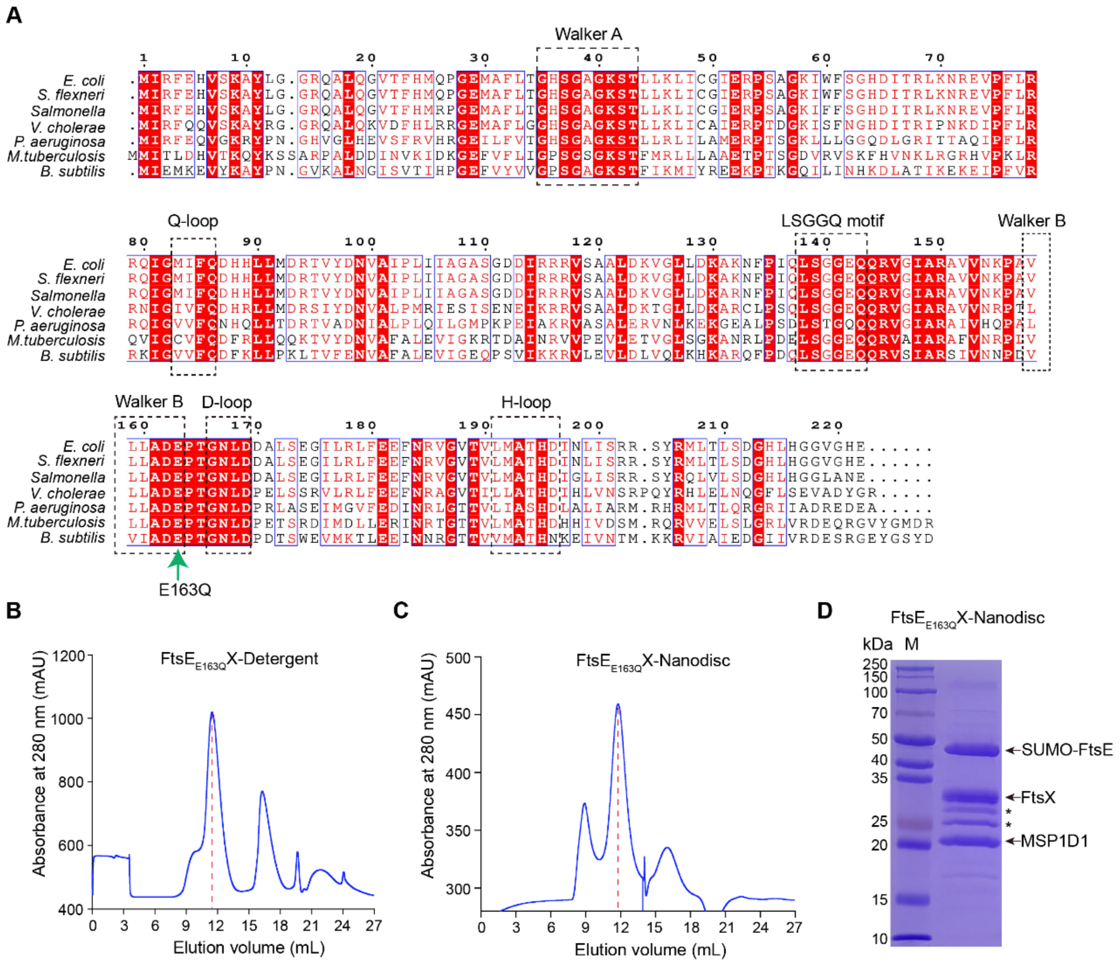

Supplementary Figure 1. Sequence alignment of FtsE and the purified FtsEX complex in nanodiscs. (A) The result of the alignment of FtsE sequences from *E. coli*, *S. flexneri*, *Salmonella*, *V. cholerae*, *P. aeruginosa*, *M. tuberculosis*, and *B. subtilis* using Clustal Omega. Residues with highly conserved residues are shown in red. Secondary structure elements are shown above the alignment based on the structure of FtsE from *E. coli*. (B-C) The gel filtration profiles of FtsE(E163Q)X purification in detergent (B) and nanodisc reconstruction (C). (D) SDS-PAGE result shows the protein bands of FtsE(E163Q)X in nanodiscs. The asterisks indicate proteins not identified.

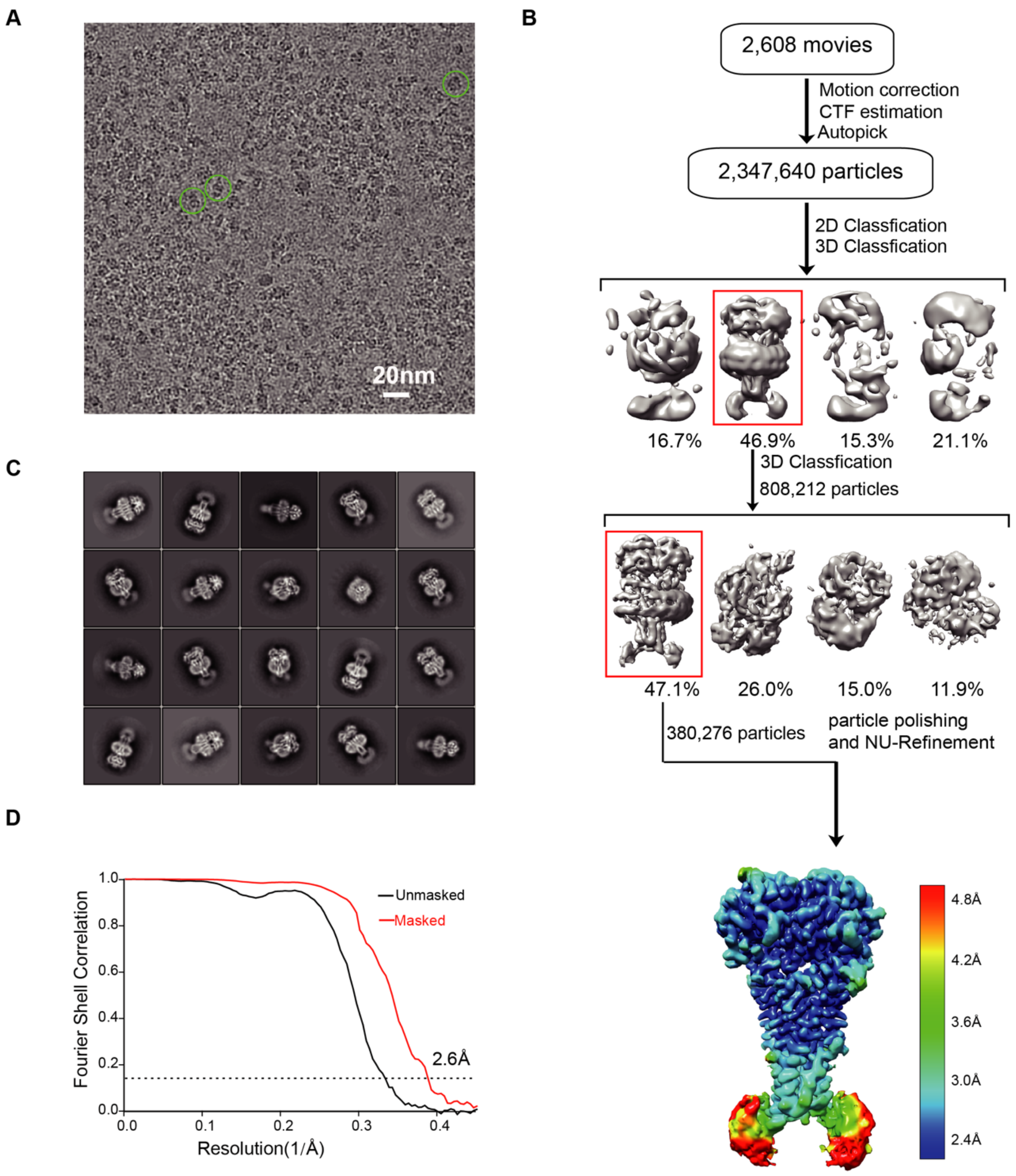

Supplementary Figure 2. Workflow of the cryo-EM image processing and 3D reconstruction for the FtsE(E163Q)X complex. (A) Representative cryo-EM microscope images of the FtsE(E163Q)X complex. (B) Scheme of data collection, two- and three-dimensional classifications, refinements and the local cryo-EM resolutions of the density maps for FtsE(E163Q)X complex. (C) Two-dimensional class averages of cryo-EM particle images FtsE(E163Q)X complex. (D) Gold-standard Fourier Shell Correction (FSC) curves for structure resolution determination.

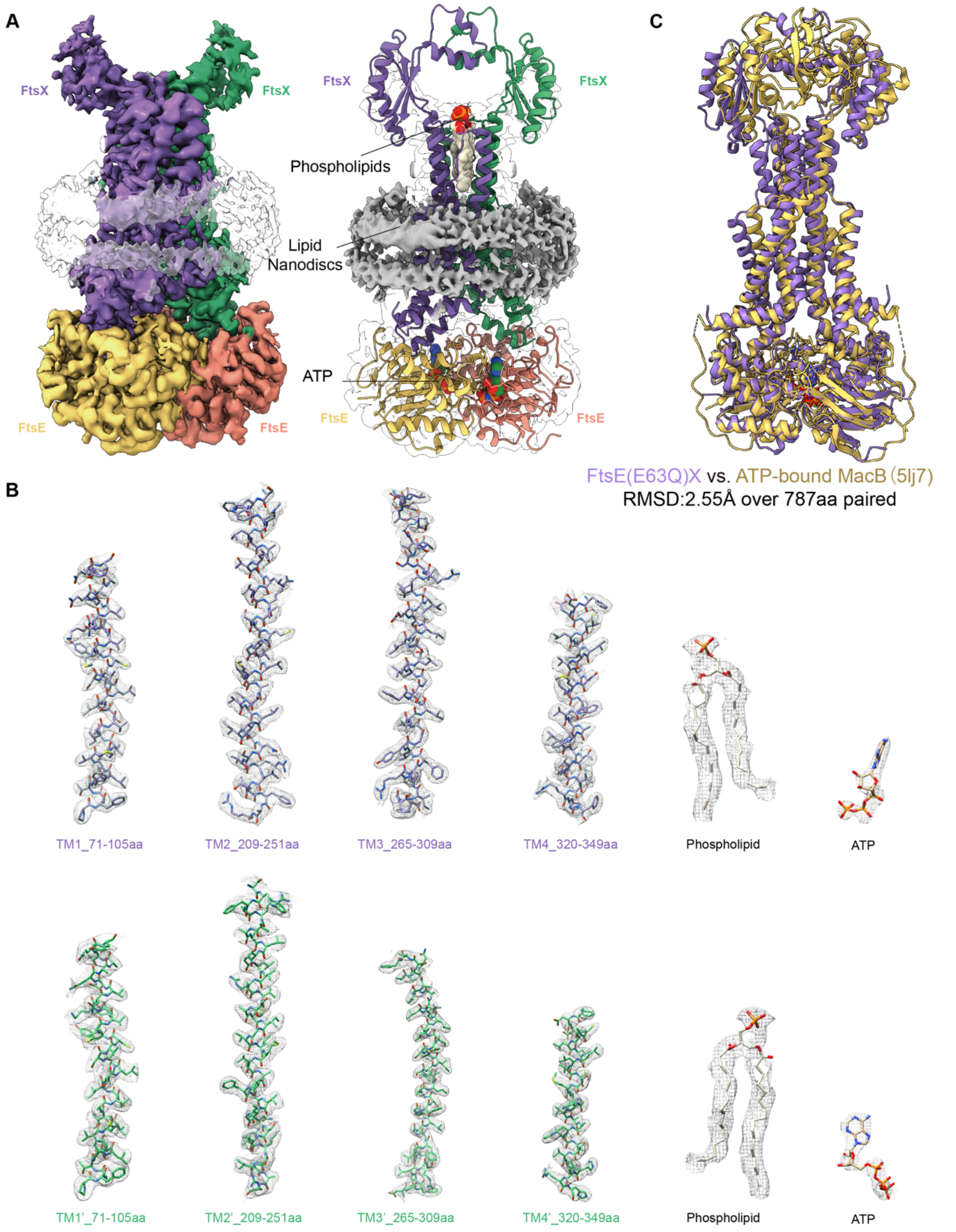

Supplementary Figure 3. The atomic model of the ATP-bound FtsEX structure fitting into its cryo-EM map densities. (A) Side view of cryo-EM map (left) and cartoon structure (right) of the ATP-bound FtsE(E163Q)X in lipid nanodiscs. (B) Cryo-EM maps and cartoon model of individual transmembrane helices of FtsX and the phospholipids. (C) Superimposition of the ATP-bound FtsEX and MacB (PDB:5lj7) with an RMSD of 2.55Å over 787 residues aligned.

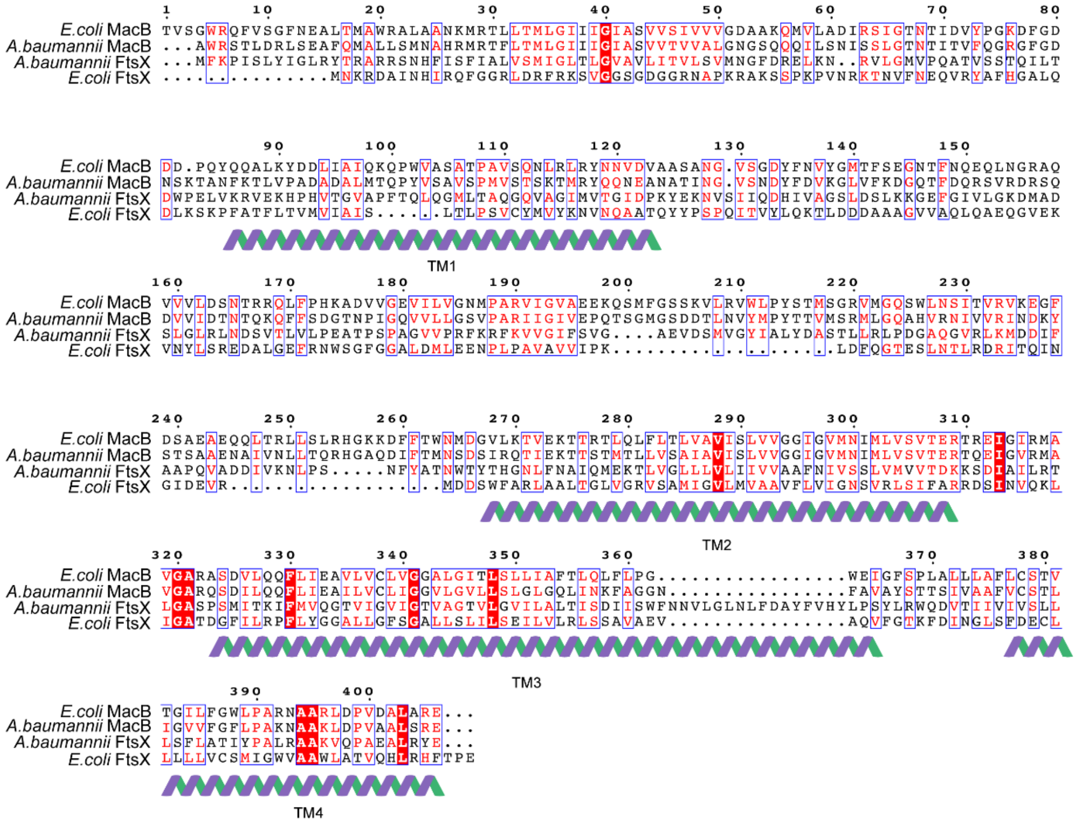

Supplementary Figure 4. Sequence alignments of the transmembrane domain (TMD) and periplasmic loop domain (PLD) of MacB and FtsX from *E. coli* and *A.baumannii*. Transmembrane segments TM1-TM4 are indicated by the wavy lines. FtsX is less conserved among different species compared to that of FtsE.

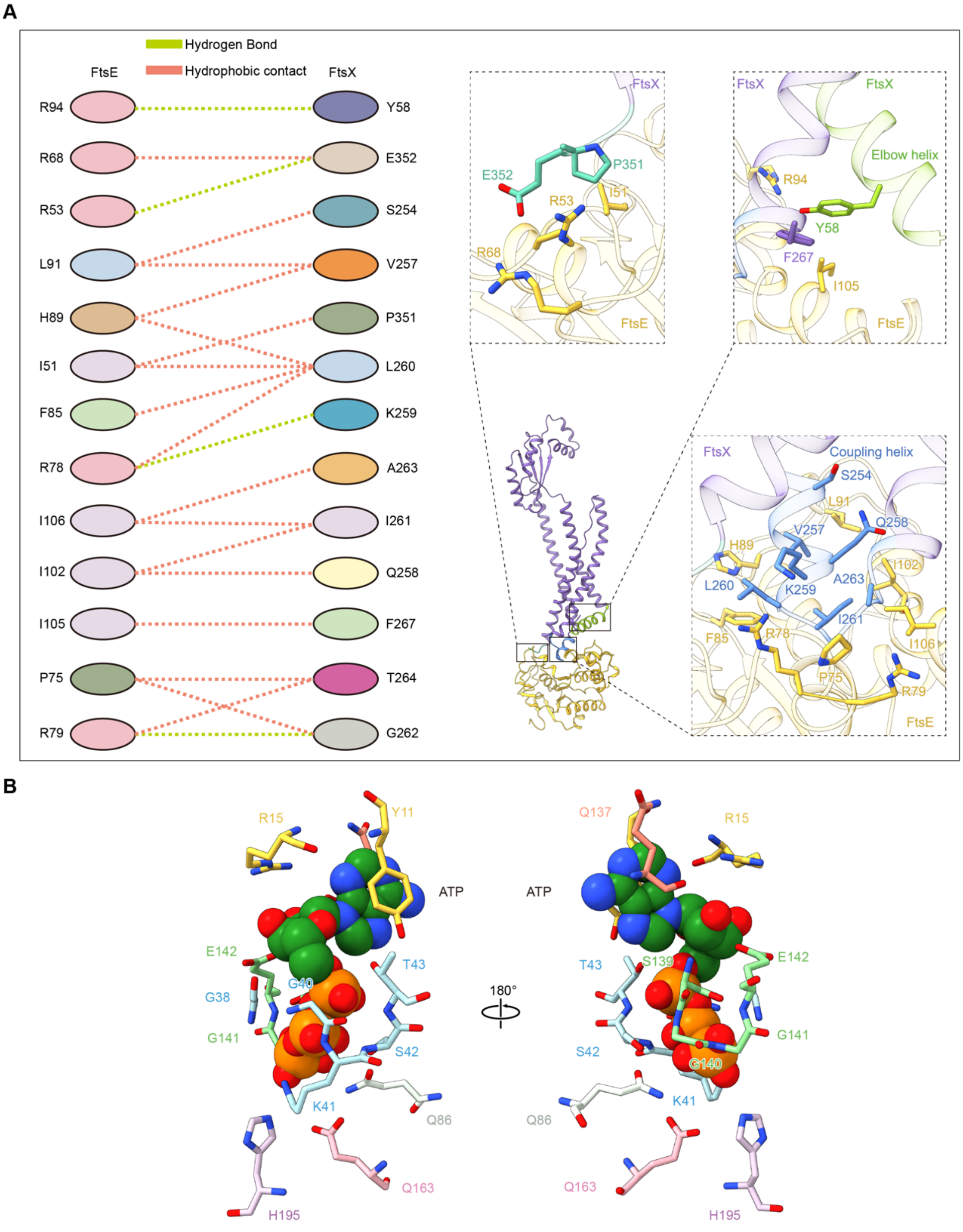

Supplementary Figure 5. Interaction between FtsE and FtsX and ATP binding pocket of FtsE. (A) Left panel: Interaction network between FtsE and FtsX. Right panel: FtsX interacts with FtsE through the elbow helix (EH) and coupling helices (CH). (B) Interactions between FtsE and the ATP molecule.

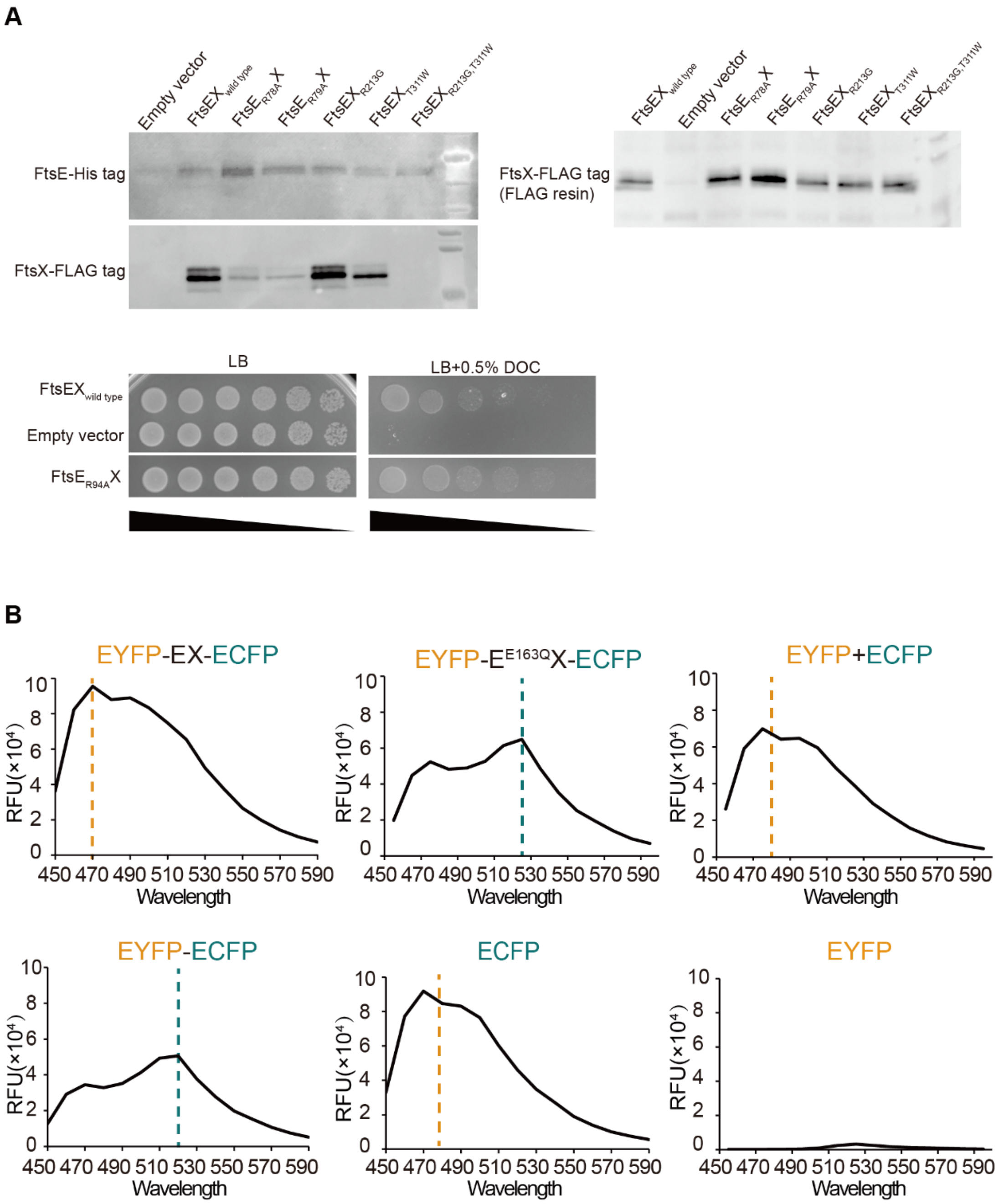

Supplementary Figure 6. Protein expression assessment of the FtsEX complex (A) Top panel: Western blot analysis of the FtsEX mutants. Top Left: The protein complex was pulled down using a nickel affinity column. The purified proteins are detected for his-tagged FtsE and Flag-tagged FtsX. Top right: The protein complex was pulled down using anti-Flag resin column and detected for the Flag-tagged FtsX. Bottom panel: Cell viability assessment of the FtsX elbow helix interactive mutant, FtsE(R94A)X. The mutation does not affect cell viability. (B) FRET assay comparing the fluorescent emission signals of the purified EYFP-FtsEX-ECFP in wild-type and FtsE(E163Q)X mutants, demonstrating a shift in fluorescent emission. These results show that FRET occurred only in the ATP-bound FtsE(E163Q)X complex, indicating that FtsE and FtsX form a stable complex, whereas the wildtype protein did not show FRET effect.

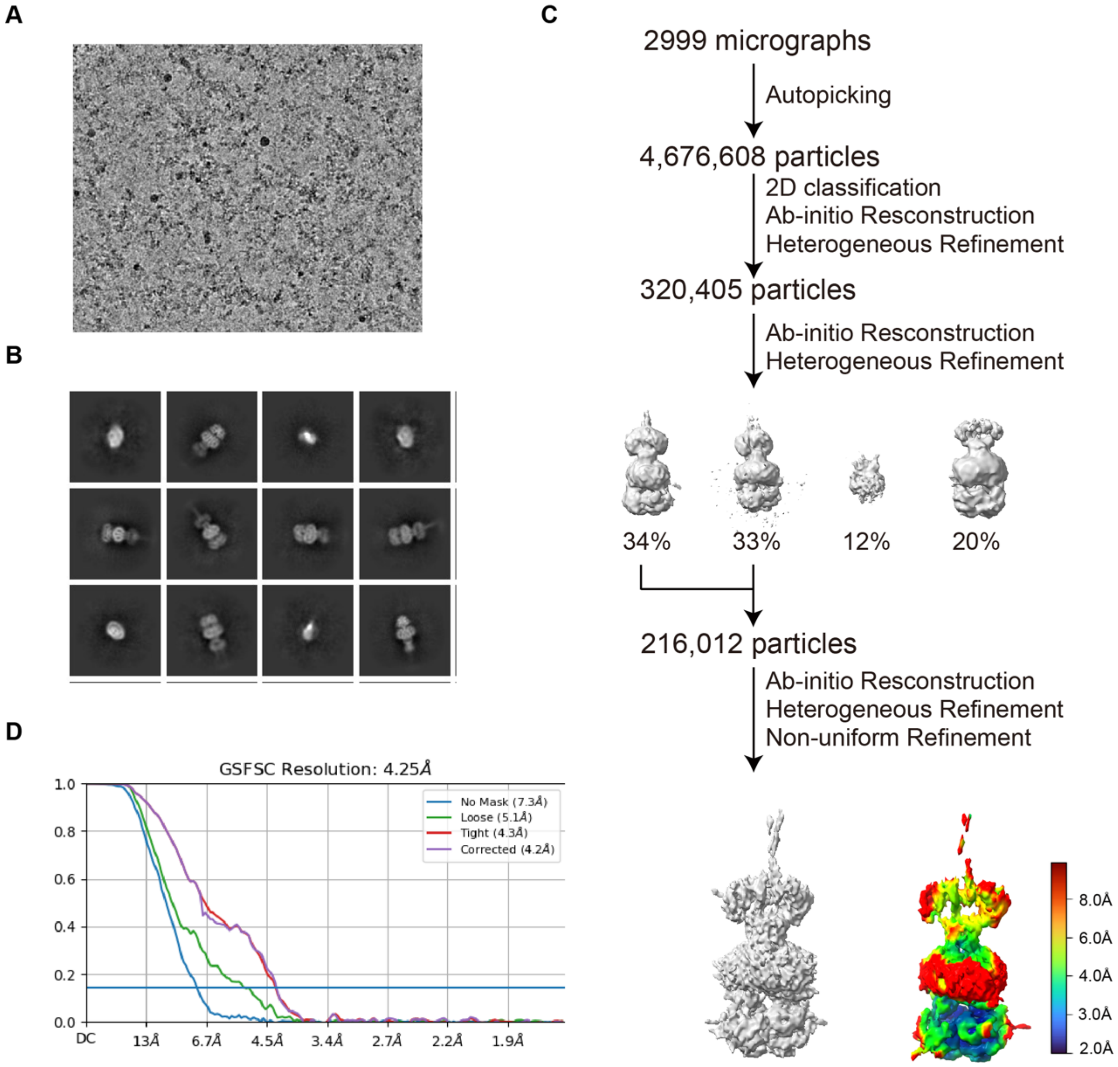

Supplementary Figure 7. Workflow of the cryo-EM image processing and 3D reconstruction for the FtsE(E163Q)X-EnvC complex. (A) Representative cryo-EM microscope images of the FtsE(E163Q)X-EnvC complex. (B) Scheme of data collection, two- and three-dimensional classifications, refinements and the local cryo-EM resolutions of the density maps for FtsE(E163Q)X-EnvC complex. (C) Two-dimensional class averages of cryo-EM particle images FtsE(E163Q)X-EnvC complex. (D) Gold-standard Fourier Shell Correction (FSC) curves for structure resolution determination.

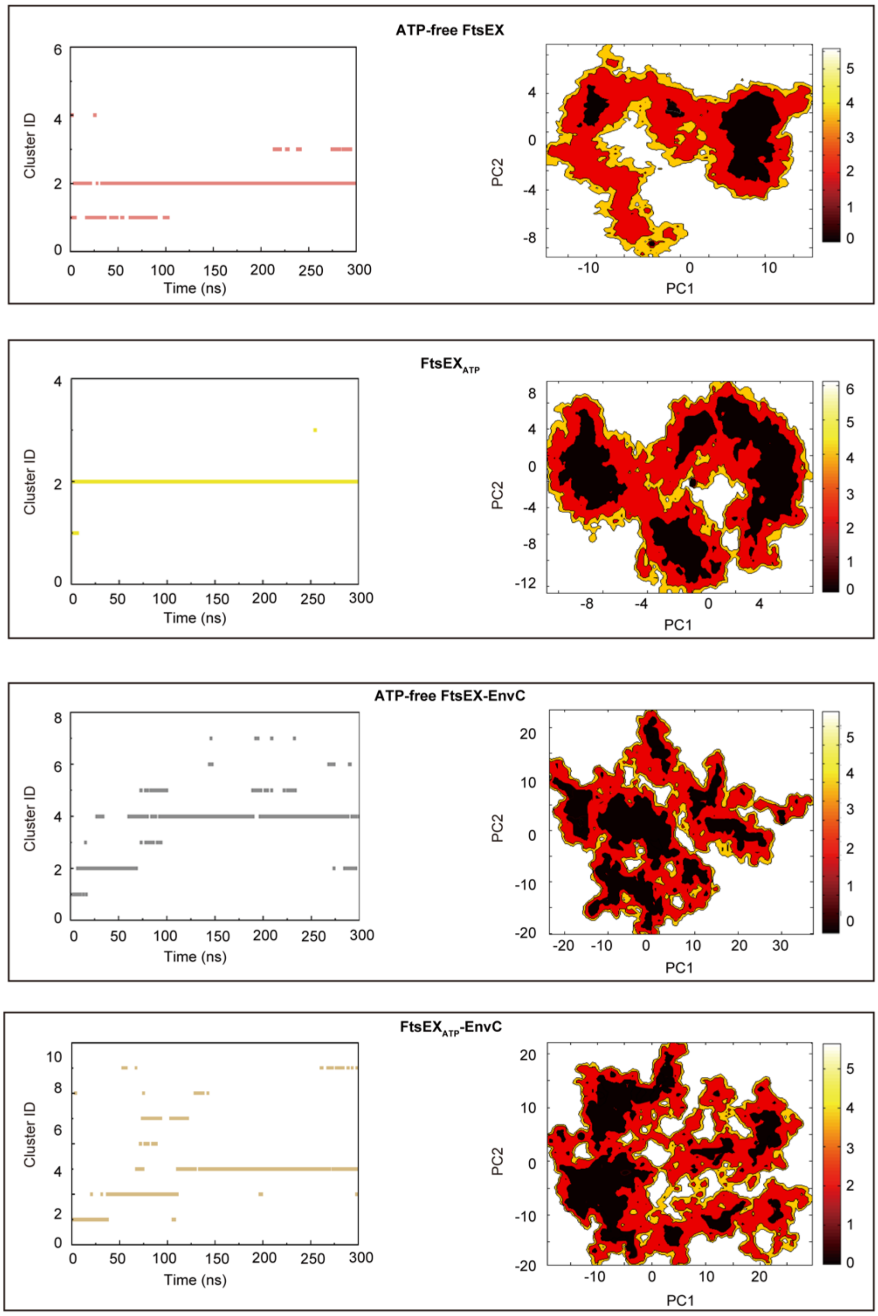

Supplementary Figure 8. Conformational cluster and free energy landscapes (FEL) analysis. Conformational cluster analysis (left) and free energy landscape (FEL) analysis (right) indicate that both FtsEX and FtsEX-EnvC structures exhibit more conformational variation in the absence of ATP, suggesting greater stability in the ATP-bound conformation.

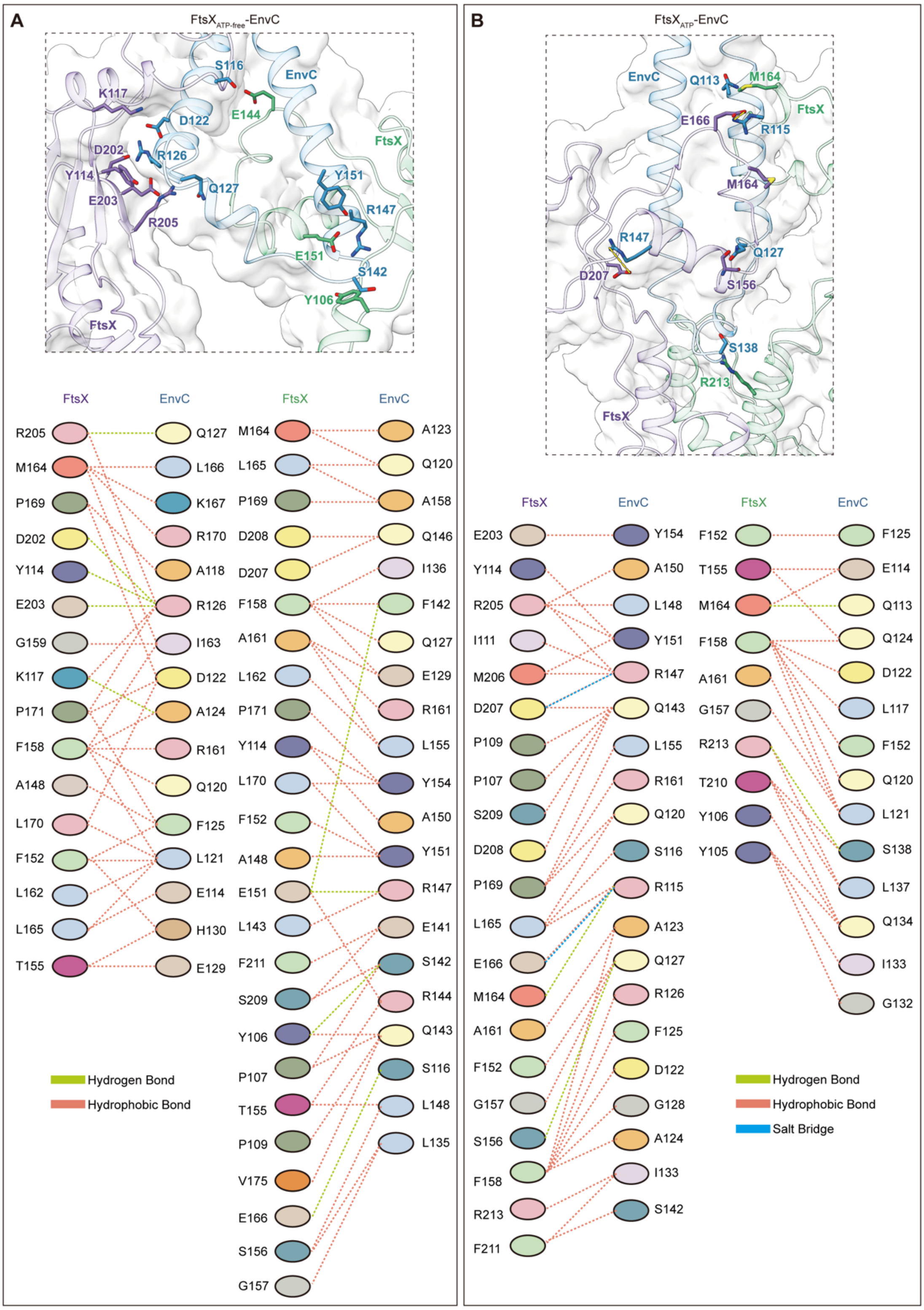

Supplementary Figure 9. Interaction networks of FtsEX and EnvC in ATP-free(A) and ATP-bound states(B). (A) Top: hydrogen bond and hydrophobic interactions of FtsX and EnvC in ATP-free state. Bottom: All interactions between FtsX and EnvC in ATP-free state. (B) Hydrogen bond, hydrophobic, and salt bridge interactions of FtsX and EnvC in ATP-bound state. Bottom: All interactions between FtsX and EnvC in ATP-bound state.

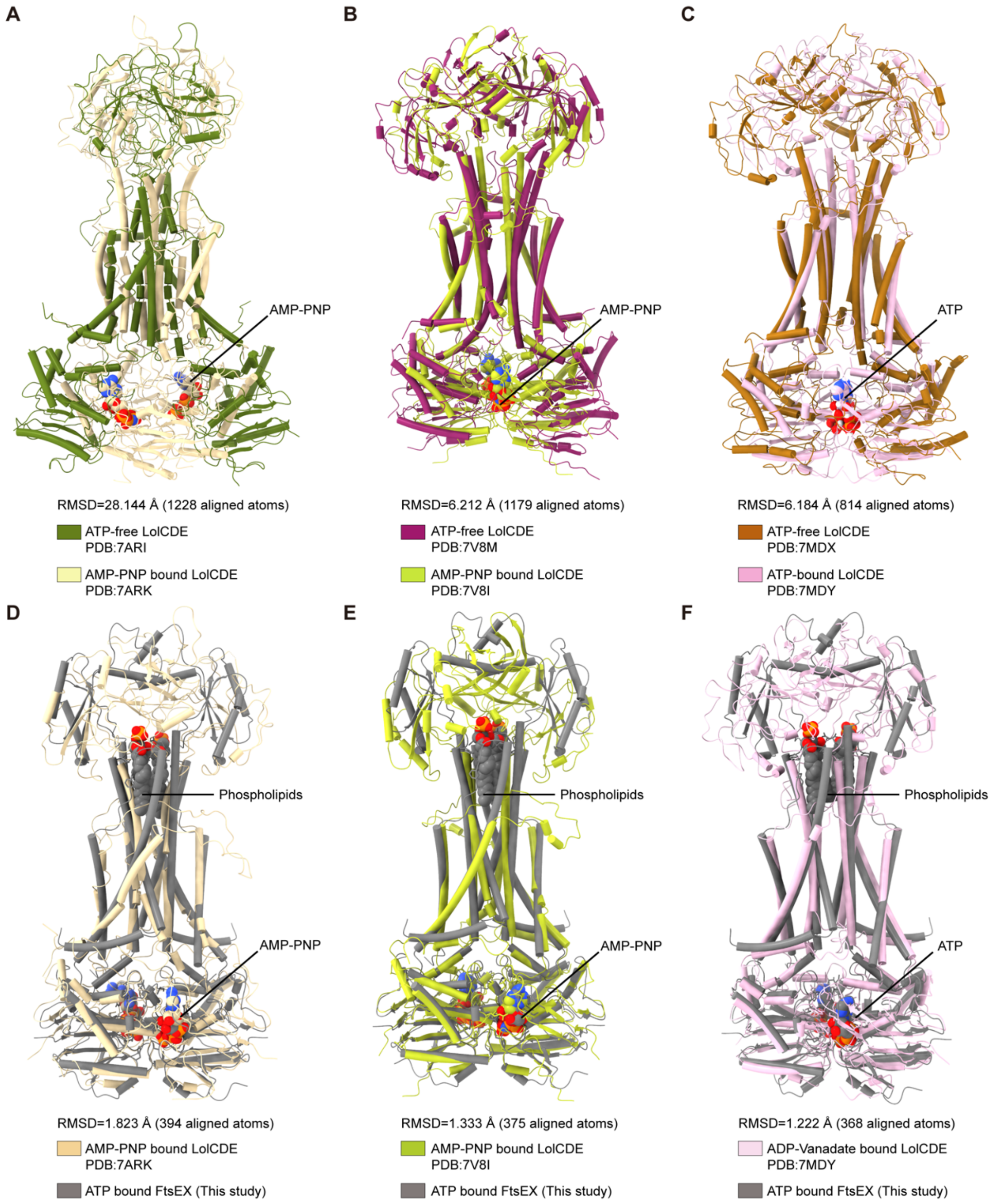

Supplementary Figure 10. Structure comparison of LolCDE from different species with our FtsE(E163QX) structure. (A) Superimpose of *E. coli* ATP-free LolCDE (PDB:7ARI) with AMP-PNP bound LolCDE(7ARK). (B) Superimpose of *E. coli* ATP-free LolCDE (PDB:7V8M) with AMP-PNP bound LolCDE(7V8I). (C) Superimpose of *E. coli* ATP-free LolCDE (PDB:7MDX) with ADP-bound LolCDE(7MDY). (D-F) Superimpose of *E. coli* ADP bound LolCDE (7MDY, 7V8I, 7MDY) with our *E. coli* FtsE(E163QX) structure.

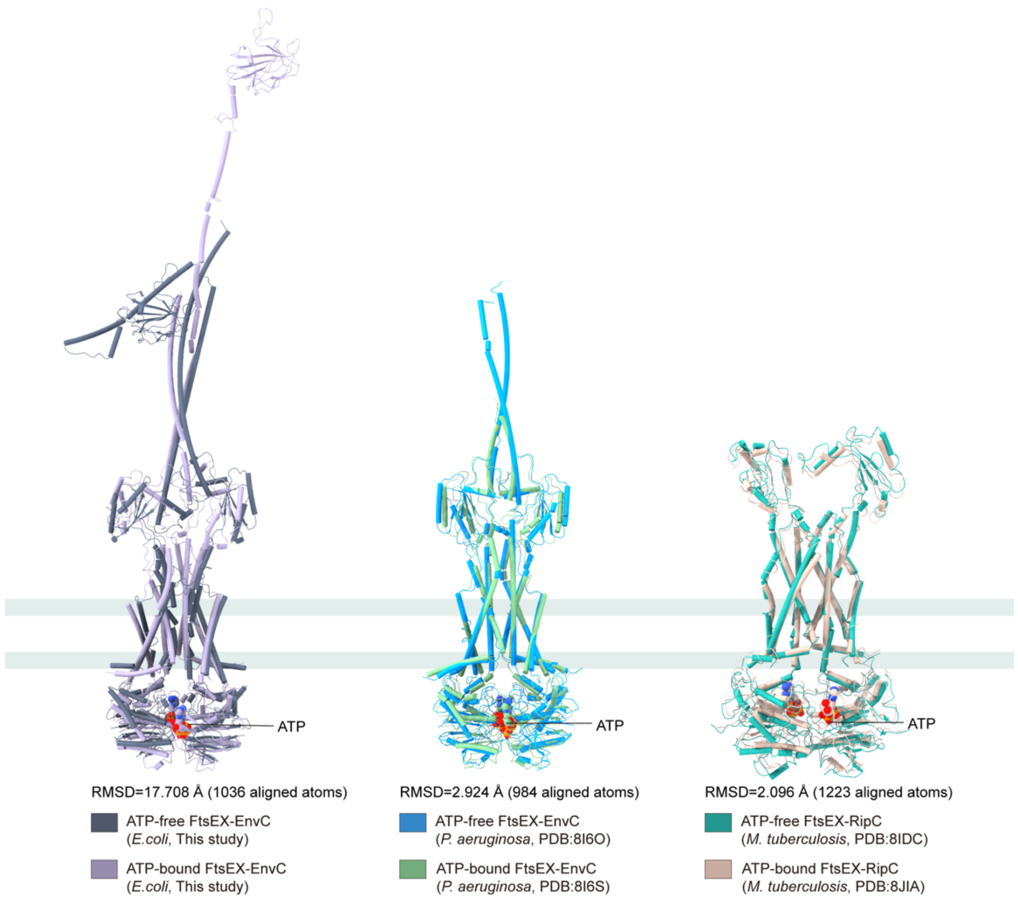

Supplementary Figure 11. Structure comparison of FtsEX-EnvC (or RipC) from various species in different states, showing similar conformational changes between states. Species include *E. coli*, *Pseudomonas aeruginosa*, and *Mycobacterium tuberculosis*.
